# A tandem simulation framework for predicting mapping quality

**DOI:** 10.1186/s13059-017-1290-3

**Published:** 2017-08-10

**Authors:** Ben Langmead

**Affiliations:** 1 Department of Computer Science, Whiting School of Engineering, Johns Hopkins University, 3400 North Charles St, Baltimore, 21218-2682 USA; 2 Department of Biostatistics, Bloomberg School of Public Health, Johns Hopkins University, 615 N Wolfe St, Baltimore, 21205 USA

**Keywords:** Sequencing, Read alignment, Mapping, Quality

## Abstract

Read alignment is the first step in most sequencing data analyses. Because a read’s point of origin can be ambiguous, aligners report a mapping quality, which is the probability that the reported alignment is incorrect. Despite its importance, there is no established and general method for calculating mapping quality. I describe a framework for predicting mapping qualities that works by simulating a set of tandem reads. These are like the input reads in important ways, but the true point of origin is known. I implement this method in an accurate and low-overhead tool called Qtip, which is compatible with popular aligners.

**Electronic supplementary material:**

The online version of this article (doi:10.1186/s13059-017-1290-3) contains supplementary material, which is available to authorized users.

## Introduction

Read alignment is often the first task when analyzing sequencing data. This is the process of determining each read’s point of origin with respect to a reference genome. Much prior work is concerned with making read aligners computationally efficient [1]. That said, a read’s point of origin can be ambiguous, and the reported alignments can be incorrect [2]. Repetitive genomes, sequencing errors, and genetic differences contribute to the problem. In addition to being efficient, aligners must accurately characterize the uncertainty associated with each alignment, as first proposed in the seminal MAQ study [2], which coined the term “mapping quality.” Aligners have methods for predicting mapping quality, which is reported in the MAPQ field of the SAM/BAM format [3]. These methods are generally quite ad hoc, and are not well described in research literature or software manuals.

We introduce the *tandem simulation* framework for predicting mapping qualities for all the alignments in a dataset in a manner that is agnostic to the aligner and parameters used. We also introduce Qtip, a tool implementing the framework. Qtip operates alongside and in cooperation with an aligner like Bowtie 2 [4]; the term “tandem simulation” refers to this cooperation. After observing the input reads and alignments, Qtip trains an ensemble tree model for predicting mapping qualities. Training uses simulated tandem reads, which are randomly drawn from the genome but crafted in a way that mimics the statistical properties of the input reads, including their length, quality, gap, and edit distributions. The aligner must be modified to report feature data for the model, but alignment algorithms need not be changed. We implemented changes for the Bowtie 2 [4], BWA-MEM [5], and SNAP [6] aligners. Qtip works with any aligner that outputs feature data in a special SAM field; it is not limited to the tools adapted for this study.

We demonstrate that Qtip’s predictions are superior to those made by the read aligners themselves, both on average and for most specific MAPQ thresholds tested. We use simulation experiments to show this for various read aligners (Bowtie 2, BWA-MEM, and SNAP), alignment settings (read lengths, alignment parameters, and species), and accuracy criteria. We also perform a variant-calling experiment to show the improved mapping qualities can benefit downstream analysis. To our knowledge, this is the first description of a general technique for characterizing alignment uncertainty that is applicable across software tools and alignment settings.

## Background

### Alignment errors

Given a sequencing read and reference genome, a read aligner like Bowtie 2 [4], BWA-MEM [5] or SNAP [6] will search for the read’s highest-scoring alignment to a substring of the reference. An alignment score measures the degree of similarity between the strings, with a higher score indicating fewer mismatches and gaps. If more than one alignment has the maximal score, one is chosen arbitrarily. Though many aligners can be configured to report more than one alignment per read, we assume here that just one is reported, as is common. If the reported alignment does not correspond to the read’s true origin, the alignment is *incorrect*, and we call this an *alignment error*. Incorrect alignments lead to interpretation problems later [7, 8].

Aligners use heuristics – computational shortcuts – to limit the effort expended. Heuristics affect which alignments can and cannot be found, shaping what errors the aligner might make. Additional file 1: Note 1 outlines the heuristics used by Bowtie 2.

We can divide alignment errors into three categories, as suggested in the MAQ study [2]:

1. The read is reported to have originated from a locus in the reference genome, but actually originates from a sequence not included in the reference.
2. No alignment to the reference is found, but the read actually originates from some locus in the reference.
3. An alignment to locus *L*_*r*_ in the reference is reported, but the read actually originates from a different locus in the reference, *L*_*t*_.

Category 1 errors might be caused by contaminating DNA, or by an inappropriate or incomplete reference genome sequence. Category 2 errors can occur when the alignment at *L*_*t*_ falls below the minimum similarity threshold (*S*_min_), or when the alignment at *L*_*t*_ is missed due to alignment heuristics. Category 3 errors are caused by a combination of repetitive DNA, sequencing errors, genetic differences, and alignment heuristics. Category 3 errors and the related idea of multi mappers, reads that align equally well to many loci, are discussed in prior studies [8, 9]. Category 3 errors are also the most numerous, making up 95.8–99.7% of the errors in our simulations (Additional file 1: Notes 2-3 and Table S1).

Here we focus on the task of predicting mapping qualities for aligned reads in light of category 3 errors. Category 2 errors are not considered, since no mapping-quality prediction is needed in those cases. Although category 1 errors affect mapping-quality prediction, we assume they are rare enough to be ignored. In principle, category 1 errors could be included in our model, e.g. by assuming a global prevalence of category 1 errors and scaling predictions accordingly, or by including contamination in the simulation.

### Mapping quality

While searching for alignments, aligners uncover information that can be used to predict whether a given alignment is correct. For instance, if the aligner discovers that a read aligns equally well to several copies of a repeat, its confidence that the selected alignment is correct will be low. If the aligner discovers that a read aligns perfectly to one locus and very poorly to a few others, confidence will be higher. Confidence is measured as the probability *p* that the reported alignment is correct. Let the *mapping quality* $q = -10 \log _{10} (1 - p)$. Higher values for *p* (or *q*) indicate higher confidence. The SAM/BAM format [3] requires that *q*, rounded to the nearest integer, be reported in the MAPQ field of each alignment. We, therefore, seek a method that predicts *q* (or equivalently, *p*) accurately across a range of alignment scenarios.

Mapping quality measures something distinct from alignment score. A high alignment score indicates high sequence similarity (few mismatches and gaps) between read and reference. It does not imply high mapping quality. For instance, consider a read that aligns with no gaps or mismatches to two distinct loci in the reference. The alignment score is high because there are no gaps or mismatches, but there is only a 50% chance of choosing the correct alignment (*q*≤3). Other measures that do not take genomic repeats into account, such as *E* values [10], are also poor proxies for mapping quality.

### Related work

The MAQ study [2] describes sources of alignment error and presents a model for predicting *q* given alignment scores for the best and second-best alignments, and the number of alignments tied for second best. Successors to MAQ, such as BWA [11], BWA-SW [12], and BWA-MEM [5], use more complex prediction functions. For example, BWA-MEM uses information about whether and how seeds – substrings of the read – match the genome. Qtip uses similar data to train its model. Qtip takes a general approach, learning the prediction model from data, and can adapt to a variety of aligners and alignment settings.

ARDEN [13] uses a mutated decoy genome to estimate the aggregate prevalence of category 3 errors. However, it is only concerned with aggregate summaries and does not predict *q* for individual alignments. LoQuM [14] uses simulated training alignments and a logistic regression model to predict new *q*’s for an already-aligned dataset. Unlike Qtip, LoQuM does not predict *q* from scratch; rather, it recalibrates *q* using the aligner-reported mapping quality as an input, along with other inputs derived from the alignment.

The MOSAIK [15] aligner uses a neural network to predict *q*. The user trains the model ahead of time, supplying simulated reads annotated with their true point of origin. Model features include alignment scores of the best and second-best alignments, read sequence entropy, and the number of potential mapping locations. Tandem simulation works like MOSAIK’s approach, building a model from simulated reads, but without requiring the user to collect training data.

Tandem simulation also has similarities to a previous method for allele-specific expression proposed by Hodgkinson et al. [16]. In that method, RNA sequencing reads are aligned to a reference genome and allelic ratios are computed at heterozygous sites. The method then simulates a null dataset where (a) the genome from which the reads are simulated is customized to include non-reference alleles detected in a separate assay and (b) when a simulated read overlaps a heterozygous variant, both alleles are sampled with equal frequency. Null reads are aligned to the original reference using the same aligner and parameters as in the initial alignment step, much like the alignment of tandem reads in our framework. Allelic ratios derived from null alignments are used to normalize the original ratios, reducing bias. While our method and Hodgkinson et al.’s target different problems, they are alike in their use of a newly simulated dataset to improve results from an initial alignment.

## Results

### Experimental conditions

Simulations were conducted using Mason v0.1.2 [17], or a different simulator where indicated. We ran Qtip v1.6.2 in combination with Bowtie 2 v2.3.2, BWA-MEM v0.7.15, and SNAP v1.0beta.18. Experiments were performed on nodes of the Maryland Advanced Research Computing Center; each node is an Intel Haswell system with two 12-core processors (2.5 GHz) and 128 GB RAM.

All read aligners were run in their default reporting modes. In other words, all aligners report up to one best alignment per read. Reads that fail to align are excluded from the analysis. We used the GRCh38 assembly with some short sequences filtered out (see Additional file 1: Note 4) as our human reference, except where otherwise noted. Qtip ran on Python v2.7.12 and used scikit-learn v0.18.

### Plots and measures

Let *A* be a vector of *n* alignments *a*_0_,*a*_1_,…,*a*_*n*−1_. Let correct(*a*_*i*_)=1 if *a*_*i*_ is correct and 0 otherwise. Let incorrect(*a*_*i*_)=1−correct(*a*_*i*_). An alignment is considered correct if the leftmost base involved in the alignment is within 30 nucleotides (nt) of the leftmost base in the simulated substring, with appropriate adjustments for soft clipping. Let *Q*=*q*_0_,*q*_1_,…,*q*_*n*−1_ be mapping qualities corresponding to *a*_0_,*a*_1_,…,*a*_*n*−1_, as predicted by the read aligner, and let *P*=*p*_0_,*p*_1_,…,*p*_*n*−1_ be the corresponding correctness probabilities, using the relationship that $q = -10 \log _{10} (1 - p)$. *Q* ^′^ and *P* ^′^ are defined similarly, but for the mapping qualities predicted by Qtip.

We define plots (cumulative incorrect difference or CID and cumulative squared-error difference or CSED) and measures (relative change in area under CID or RCA, and relative change in sum of squared errors or RCE) that characterize how Qtip’s predictions (*Q* ^′^) compare to the aligner’s (*Q*). CID and RCA capture how well *Q* ^′^ *ranks* alignments from most to least likely to be correct relative to *Q*. CID and RCA are invariant under monotonic transformations of *P* and *P* ^′^; they are concerned only with how well alignments are ranked, not with probabilities per se. CSED and RCE capture how closely *P* ^′^ matches the the true correctness relative to *P*; i.e., CSED and RCE are concerned with how well *P* ^′^ and *P* fit their probabilistic interpretation.

#### Cumulative incorrect difference

Let $\hat {A}$ be *A* sorted in descending order by *Q*, and likewise for $\hat {A}'$ and *Q* ^′^. The *cumulative incorrect vector* *C* is the vector *c*_0_,*c*_1_,…,*c*_*n*−1_ such that $c_{i} = {\sum \nolimits }_{j=0}^{i} \text {incorrect}(\hat {a}_{j}).$ ^1^ *C* ^′^ is defined similarly for $\hat {A}'$. Let *D* be the element-wise difference *C* ^′^−*C*. When *d*_*i*_<0, Qtip’s mapping qualities yield a better segregation of correct from incorrect alignments about the pivot *i*. When *d*_*i*_>0, the aligner’s mapping qualities give the better segregation. A CID plot draws a line representing the *d*_*i*_’s (vertical axis) for *i*=0 to *n*−1 (horizontal axis), and we judge Qtip’s efficacy according to the line’s tendency to stay below *y*=0.

#### Cumulative squared-error difference

Let $\hat {A}$ and $\hat {P}$ be *A* and *P* sorted in descending order by *P*, and likewise for $\hat {A}'$ and $\hat {P}'$. The *cumulative squared error vector* *E* is the vector *e*_0_,*e*_1_,…,*e*_*n*−1_ such that $e_{i} = {\sum \nolimits }_{j=0}^{i} (\text {correct}(\hat {a}_{j}) - \hat {p}_{j})^{2}$, with *E* ^′^ defined similarly for $\hat {A}'$ and $\hat {P}'$.^2^ Let *S* be the element-wise difference *E* ^′^−*E*. When *s*_*i*_<0, Qtip’s mapping qualities yield a lower squared error up to the *i*th alignment.

The CSED plot draws a line representing the *s*_*i*_’s (vertical axis) for *i*=0 to *n*−1 (horizontal axis). Like for the CID plot, we judge Qtip’s efficacy according to the line’s tendency to stay below *y*=0.

#### Relative change in area under CID

RCA is defined as $\left (\sum _{i=0}^{n-1} c'_{i} - \sum _{i=0}^{n-1} c_{i}\right)/ \sum _{i=0}^{n-1} c_{i}$. Negative values indicate that a better overall ranking is achieved using Qtip’s predictions.

#### Relative change in sum of squared errors

RCE is defined as (SSE(*P* ^′^)−SSE(*P*))/SSE(*P*), where $\text {SSE}(P) = \sum _{i=0}^{n-1} (\text {correct}(a_{i}) - p_{i})^{2}$. Negative values indicate that Qtip’s predictions yield a lower total squared error.

The distinction between the rank-based (CID and RCA) and probabilistic (CSED and RCE) metrics relates to how downstream tools, e.g. variant callers, use mapping qualities. Freebayes [18] and the Genome Analysis Toolkit (GATK) [19] ignore an alignment if its mapping quality is below a threshold. In this case, CID and RCA are relevant as they directly evaluate how well various thresholds separate correct from incorrect alignments. Other methods, such as the consensus genotype calling method described in the MAQ study [2], interpret a mapping quality as a probability. Alignments are weighted according to their probability, with no alignments excluded. Here, CSED and RCE are relevant since they directly evaluate how well the probabilities match the actual correctness status.

We note that the problem of evaluating and plotting the relative quality of two sets of mapping-quality predictions is not specifically addressed in past studies. Receiver operating curve (ROC)-like plots are used for the related task of comparing aligners [4, 5], where the axes represent false and true positives and a line follows points corresponding to increasingly permissive mapping-quality thresholds. However, the two-dimensionality of these plots makes it hard to find comparable points, that is points on two curves where the threshold allows same number of alignments. A similar problem exists for comparisons examining particular thresholds (≥10, ≥20, etc.); for two sets of predictions, the thresholds might allow very different numbers of alignments, impeding interpretation. CID and CSED plots are inspired by accuracy versus drop rate plots [20] and are related to ROC-like plots, except (a) two lines are represented more concisely as a single line giving the difference and (b) at a given horizontal point, we are comparing thresholds that allow the same number of alignments (the same drop rate).

### Simulation experiments

We conducted simulation experiments to show how Qtip’s mapping-quality predictions compare to those made by the read aligners. We vary several experimental conditions, including (a) read length, (b) aligner parameterization, (c) reference genome, (d) read alignment tool, and (e) read simulator. The simulator encodes the read’s true point of origin in the read name, allowing Qtip to check later whether an alignment is correct.

### Simulated samples

We used Mason to simulate five Illumina-like samples with unpaired reads of length 50, 100, 150, 250, and 500, respectively. We simulated five paired-end samples with the same lengths, with most fragment lengths being between 2*L* and 4*L* nt, where *L* is the read length. We simulated 4 million reads/pairs for each sample. Aligners were configured to consider fragment lengths in the 2*L*– 4*L* range as concordant. Thus, most simulated pairs aligned concordantly (consistent with paired-end constraints) whereas some aligned discordantly. Simulator commands, and implications for fragment lengths, are discussed in Additional file 1: Note 4. Alignment commands are in Additional file 1: Note 5.

### Varying read length

**Fig. 1:**
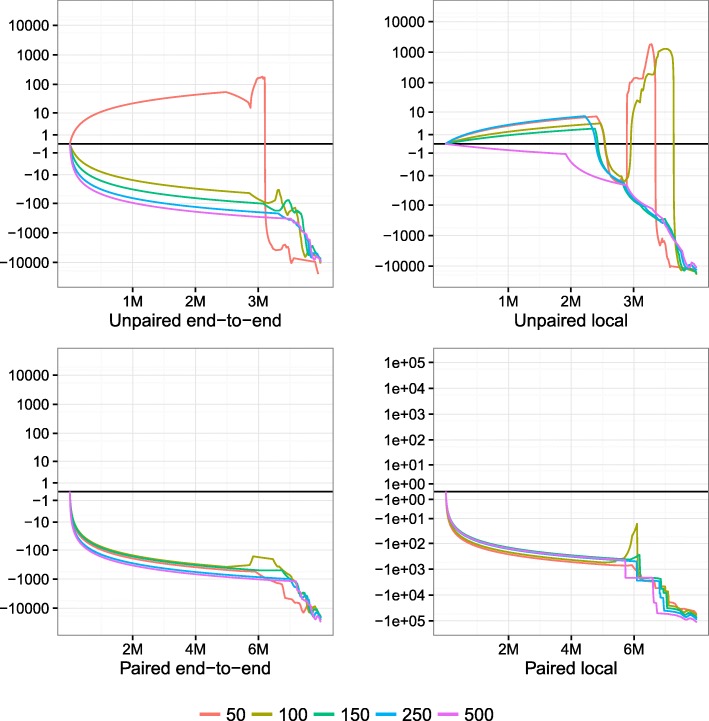
CSED for various lengths. Cumulative squared-error difference plot from running Qtip and Bowtie 2 on Mason-simulated Illumina-like samples of various lengths. Each sample consists of 4 million reads or pairs. The *horizontal axis* indicates the cumulative number of reads/ends passing the threshold, with the *left-hand extreme* corresponding to a high mapping-quality threshold and the *right-hand extreme* corresponding to a low threshold. Results for unpaired samples are on *top*, paired on *bottom*. Bowtie 2 is run in its (default) end-to-end alignment mode for the left-hand plots, and in local alignment mode for the right-hand plots. *CSED* cumulative squared-error difference

We used Qtip together with Bowtie 2 to align and predict mapping qualities for each Mason-simulated sample. We rounded Qtip’s predictions to the nearest integer per the SAM/BAM format. For each alignment, we parsed aligner-predicted and Qtip-predicted mapping qualities, as well as the read’s true point of origin as provided by Mason. We calculated RCA and RCE (Table 1) and plotted CSED (Fig. 1). CSED *y* values were scaled with $y_{\text {plot}} = \text {sign}(y_{\text {orig}}) \log _{10}(|y_{\text {orig}}|+1)$.

**Table 1:** Relative change in area under CID (RCA) and relative change in sum of squared error (RCE) when running Qtip and Bowtie 2 on Mason-simulated Illumina-like samples of various lengths

		End-to-end				Local			
		RCA		RCE		RCA		RCE	
	Read length	Mean	SD	Mean	SD	Mean	SD	Mean	SD
Unpaired	50	−9.03	0.26	−24.61	0.19	−2.49	0.47	−15.30	0.57
	100	−7.43	1.82	−18.53	2.61	−10.26	1.52	−27.87	1.59
	150	−9.11	1.15	−16.22	0.62	−14.77	2.15	−29.27	1.03
	250	−16.82	2.01	−19.97	0.44	−22.51	1.77	−28.85	0.62
	500	−33.77	0.60	−27.26	0.37	−37.75	1.32	−31.65	1.01
Paired	50	−13.11	0.44	−19.60	0.44	−18.60	0.28	−33.63	0.24
	100	−15.80	1.29	−21.79	0.33	−36.84	0.69	−45.42	0.53
	150	−22.39	1.74	−25.94	0.28	−46.87	0.54	−52.76	0.56
	250	−37.65	0.97	−33.48	0.22	−58.39	0.91	−58.75	1.38
	500	−54.59	0.58	−44.91	0.34	−68.54	0.95	−70.37	3.45

Relative change is expressed as a percentage. Each sample consists of 4 million reads/pairs. Samples are either unpaired or paired-end, and Bowtie 2 is run in either end-to-end or local alignment mode as indicated. Results are means and standard deviations over ten random trials, repeated starting from the input modeling step

*CID* cumulative incorrect difference

*RCA* relative change in area under CID

*RCE* relative change in sum of squared errors

*SD* standard deviation

To measure the variability of Qtip’s predictions, we repeated each experiment ten times starting from step 2 onward, seeding the pseudo-random number generator differently in each trial. RCA and RCE tables describe all ten trials whereas, for clarity, the CSED plot describes only the first trial.

Qtip’s mapping qualities are, overall, superior to those predicted by Bowtie 2, as indicated by the negative RCAs and RCEs (Table 1). This is true across all samples tested, and in both end-to-end and local alignment mode. The improvement is larger for samples with longer reads and for paired-end samples. Variability is modest overall but somewhat higher for longer reads. See “Discussion” for further comments on variability.

There are portions of the CSED plots (Fig. 1) where the plot rises above *y*=0, indicating the aligner-reported mapping qualities exhibit a lower cumulative squared error at those thresholds. This is most prominent in the unpaired experiments, particularly for 50 nt reads. However, Qtip’s superior predictions at other *q* thresholds – especially low ones – help bring the overall RCE below zero in all cases. For paired-end samples, CSEDs show Qtip’s predictions are superior at nearly all *q* thresholds.

### Varying the reference genome and alignment tool

To study how genomes of varying length and repetitiveness influence Qtip’s performance, we experimented with four reference genome assemblies spanning three species: human GRCh37, human GRCh38, mouse GRCm38, and *Zea mays* AGPv4. The human GRCh38 primary assembly is 3.10 Gbp long (2.95 Gbp excluding N’s) with 50% of the genome annotated as repetitive according to RepeatMasker [21]. GRCh37 is 3.10 Gbp long (2.86 Gbp excluding N’s) with 47% of the genome annotated as repetitive. GRCm38 is 2.73 Gbp long (2.65 Gbp excluding N’s), with 44% of the genome annotated as repetitive. AGPv4 is 2.13 Gbp long (2.10 Gbp excluding N’s). Though no official RepeatMasker annotation is available, past studies report that 85% of the genome consists of transposable element sequences [22], making it the most repetitive of the genomes tested. We used the Mason-simulated 100 and 250 nt samples, both unpaired and paired-end.

We tested three aligners – Bowtie 2, BWA-MEM, and SNAP – with each genome. The changes made to each aligner for it to work with Qtip are detailed in Additional file 1: Note 6. We calculated RCA and RCE for the ten trials and plotted CSED for only the first trial.

Qtip-predicted mapping qualities are superior in nearly all scenarios, as indicated by negative RCAs and RCEs (Table 2). The exceptions are three of the human paired-end SNAP experiments (GRCh37 100 nt, GRCh37 250 nt, and GRCh38 250 nt), which have negative RCA but positive RCE. The variability of RCAs and RCEs across trials is generally modest, but tool dependent, with SNAP exhibiting the highest variabilities. BWA-MEM’s standard deviations are small, all below 0.6. Bowtie 2’s range up to 2.61 and SNAP’s up to 4.44. See “Discussion” for further comments on variability.

**Table 2:** Relative change in area under CID (RCA) and relative change in sum of squared error (RCE) for various aligners and reference genomes, expressed as percentage change

			100 nt				250 nt			
			RCA		RCE		RCA		RCE	
			Mean	SD	Mean	SD	Mean	SD	Mean	SD
Unpaired	GRCh37	Bowtie 2	−11.22	1.08	−24.43	0.66	−15.02	0.39	−28.37	0.38
		BWA-MEM	−14.49	2.29	−49.54	0.43	−9.14	2.09	−52.31	0.38
		SNAP	−15.94	0.32	−36.86	0.23	−9.53	3.88	−28.57	0.47
	GRCh38	Bowtie 2	−7.43	1.82	−18.53	2.61	−16.82	2.01	−19.97	0.44
		BWA-MEM	−15.49	0.58	−47.42	0.37	−15.78	0.57	−51.14	0.31
		SNAP	−19.58	0.18	−36.58	0.47	−14.74	0.27	−25.47	0.32
	Mouse	Bowtie 2	−5.60	0.24	−17.19	0.45	−7.05	0.33	−17.73	0.37
		BWA-MEM	−13.50	0.15	−46.25	0.27	−16.39	0.38	−51.12	0.30
		SNAP	−9.02	0.17	−31.07	0.33	−10.61	0.20	−31.78	0.43
	*Zea mays*	Bowtie 2	−6.63	0.32	−19.56	0.25	−17.09	0.38	−25.89	0.44
		BWA-MEM	−19.26	0.11	−58.32	0.26	−25.14	0.19	−66.76	0.23
		SNAP	−13.02	0.24	−38.48	0.53	−24.01	0.43	−53.80	0.42
Paired	GRCh37	Bowtie 2	−25.86	0.33	−30.26	0.50	−36.31	2.16	−38.29	0.60
		BWA-MEM	−13.33	0.23	−45.70	0.27	−10.08	0.75	−47.58	0.31
		SNAP	−56.53	1.99	1.39	2.34	−42.89	7.63	13.17	3.95
	GRCh38	Bowtie 2	−15.80	1.29	−21.79	0.33	−38.22	0.31	−33.63	0.30
		BWA-MEM	−14.19	0.16	−41.35	0.19	−12.36	0.46	−42.78	0.29
		SNAP	−51.36	0.98	−11.16	1.12	−51.32	1.45	4.34	2.29
	Mouse	Bowtie 2	−10.10	0.26	−18.93	0.31	−19.03	0.21	−29.07	0.32
		BWA-MEM	−11.86	0.12	−36.18	0.37	−13.30	0.19	−39.91	0.21
		SNAP	−29.90	0.67	−17.04	0.76	−30.16	0.30	−15.79	0.47
	*Zea mays*	Bowtie 2	−17.92	0.21	−26.95	0.27	−43.19	0.18	−51.69	0.38
		BWA-MEM	−17.04	0.15	−47.48	0.29	−21.45	0.20	−56.58	0.08
		SNAP	−36.28	0.55	−17.08	0.79	−26.45	4.44	−20.05	0.52

The experiments used 100 or 250 nt reads, and unpaired or paired-end reads, as indicated. Results are means and standard deviations over ten random trials, repeated starting from the input modeling step

*CID* cumulative incorrect difference

*RCA* relative change in area under CID

*RCE* relative change in sum of squared errors

*SD* standard deviation

**Fig. 2:**
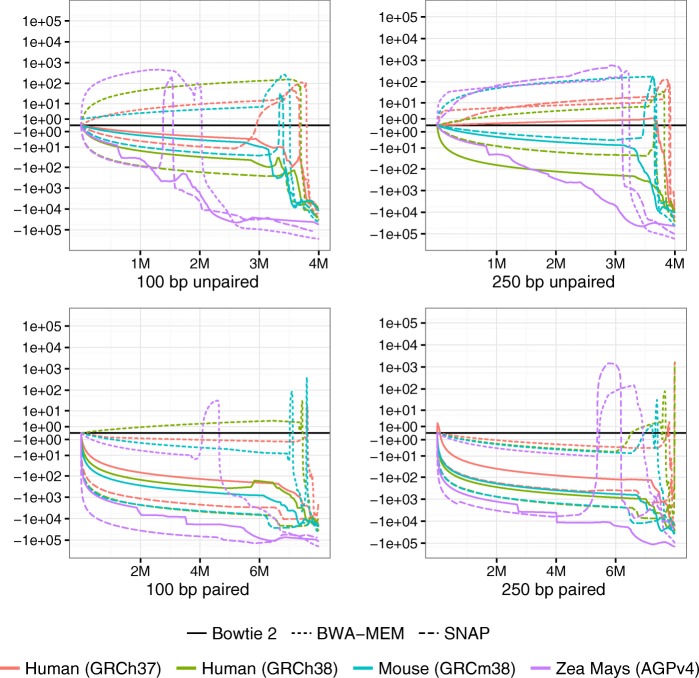
CSED for various aligners and references. Cumulative squared-error difference plot from running Qtip with various reference genomes and read aligners. The input reads are Mason-simulated Illumina-like 100 nt (*left*) and 250 nt (*right*) samples, each consisting of 4 million reads/pairs. The *horizontal axis* indicates the cumulative number of reads/ends passing the threshold, with the *left-hand extreme* corresponding to a high mapping-quality threshold and the *right-hand extreme* corresponding to a low threshold. Results for unpaired samples are shown on *top*, and paired on *bottom*. *CSED* cumulative squared-error difference

CSED curves (Fig. 2) again show that for some thresholds, aligner-reported mapping qualities are superior in terms of minimizing the cumulative squared error, i.e., where the CSED rises above *y*=0. Qtip’s mapping qualities seem to perform worse for many thresholds in the BWA-MEM unpaired experiments, especially for *Zea mays*. However, Qtip’s qualities consistently perform better at very low thresholds. Qtip’s mapping qualities perform particularly well for the Bowtie 2 *Zea mays* experiments, and for all the paired-end experiments.

To assess how the greater incidence of category 1 errors affects the results, we repeated the human experiments, expanding the simulation to include reads both from the reference genome and from sequences in the CHM1 hydatidiform mole assembly not present in the reference. We used Assemblytics [23] to obtain CHM1-specific sequences as detailed in Additional file 1: Note 3. The results show this has little effect on the accuracy of Qtip’s predictions (Additional file 1: Table S2).

### Other simulation experiments

We also conducted simulation experiments varying the sensitivity level of the aligner (described in Additional file 1: Note 7, Table S3, and Figure S1) and varying the software tool used to generate the simulated reads (described in Additional file 1: Note 8, Table S4, and Figure S2).

### Variant calling

To demonstrate Qtip’s effect on downstream results, we evaluated variant-calling accuracy with and without Qtip’s predictions. We used paired-end human 100×100 Illumina HiSeq reads from the Platinum Genomes project [24] (ERR194147) and gold-standard Platinum variants [24] for the sequenced individual (NA12878). The Platinum variants are high-confidence pedigree-validated calls supported by multiple bioinformatics pipelines and sequencing technologies. The analysis is limited to areas of the genome called with high confidence by Platinum Genomes.

We used Freebayes v1.1.0 [18] to call single-nucleotide variants (SNVs) once for the alignments with the original mapping qualities and again for the same alignments but with Qtip-predicted mapping qualities. Following past studies [25], we filtered out variant calls with read depth greater than four Poisson standard deviations above the mean. We defined a true positive as an SNV call made from ERR194147 data that matched a Platinum call, a false positive as a call made from ERR194147 that did not match any Platinum call, and a false negative as a Platinum call that did not match any ERR194147 call. We calculated *F*_*β*_ for various *β*’s. *F*_1_ (*β*=1) is the typical *F*1 score, related to the harmonic mean of precision and recall. Setting *β*>1 gives recall more weight than precision and setting *β*<1 gives precision more weight than recall. We tried values of *β* ranging from 0.25 to 4 to cover a range of precision–recall tradeoffs. Further details on alignment and variant calling are given in Additional file 1: Note 9.

Like other variant callers and downstream tools, Freebayes uses thresholds for mapping quality (*Q*) to eliminate some alignments prior to variant calling, eliminating alignments with *Q*<1 by default. Since we are concerned with the overall accuracy of mapping qualities and not with any particular threshold, we reran Freebayes with various integer *Q* thresholds: 0–12, 15, 20, and 30. Freebayes also associates a genotype quality value with each called variant, given in the VCF file’s QUAL field. We used the vcfroc tool from vcflib (https://github.com/vcflib/vcflib) to evaluate all possible QUAL thresholds for all possible *Q* thresholds, ultimately selecting *Q* and QUAL thresholds maximizing *F*_*β*_.

The results are presented in Table 3. For all *β*’s examined except the lowest (*β*=0.25), Qtip-predicted mapping qualities yielded superior *F*_*β*_. For *β*≥1, Qtip’s predictions yielded more true positives and fewer false positives than the original predictions. For 0.25≤*β*≤0.5, Qtip’s predictions yielded around 15,000–20,000 more true positives at the cost of around 300–1,500 more false positives. Notably, these improvements were achieved simply by changing the mapping qualities; the alignments are the same and the variant caller has not been modified or tuned in any way. We also note that Qtip’s improved performance is obtained using a smaller range of mapping-quality values. Qtip-predicted mapping qualities in this experiment ranged from 0 to 36, whereas Bowtie 2 mapping qualities ranged from 0 to 42.

**Table 3:** Single-nucleotide variant (SNV) *F*_*β*_ scores for various *β*’s with original mapping qualities and with Qtip-generated qualities

	Original			Qtip			*Δ* (Qtip − Orig)		
*β*	*F*_*β*_	QUAL	*Q*	*F*_*β*_	QUAL	*Q*	*F*_*β*_	TP	FP
0.250	0.9925	194	2	0.9924	213	2	-2.2e-05	+20,189	+1505
0.333	0.9906	150	3	0.9908	178	2	+2.6e-04	+16,001	+911
0.500	0.9872	87	3	0.9881	125	3	+8.3e-04	+15,143	+311
0.750	0.9843	10.6	3	0.9854	70.1	4	+1.1e-03	-413	-6537
1.000	0.9835	0.0158	3	0.9845	13.6	4	+9.4e-04	+3999	-2745
1.500	0.9846	1.79e-06	3	0.9856	0.000675	5	+1.0e-03	+4392	-1832
2.000	0.9860	5.06e-08	3	0.9870	1.38e-05	5	+1.0e-03	+3110	-5692
3.000	0.9880	1.16e-09	3	0.9889	8.64e-08	4	+8.6e-04	+2583	-7937
4.000	0.9892	1.06e-10	3	0.9899	6.58e-09	4	+7.1e-04	+1891	-13,600

Paired-end reads from ERR194147, a female, were aligned with Bowtie 2 together with Qtip. SNV variants were called with Freebayes for chromosomes 1–22 and X. Variant-quality (QUAL) and mapping-quality (*Q*) thresholds yielding the greatest *F*_*β*_ score are reported. Platinum variants were used as the true callset. Before calculating *F*_*β*_, calls outside Platinum Genomes high-confidence regions were excluded. The three rightmost columns show differences in *F*_*β*_, the number of true positive SNVs, and the number of false positive SNVs

*FP* false positive

*SNV* single-nucleotide variant

*TP* true positive

### Efficiency and overhead

The tandem simulation framework adds an overhead to the alignment process. We measured Qtip’s overhead when analyzing public datasets ERR050082 and ERR050083. Specifically, we measured how the running time and the peak memory footprint grew when Qtip ran alongside the aligner, versus when the aligner ran by itself. The running-time overhead is modest for Bowtie 2 and BWA-MEM, ranging from 5 to 10% (Table 4). For SNAP, the running-time overhead is larger, 12 to 14% for unpaired and 23 to 28% for paired-end alignment. The peak memory footprint added by Qtip was 200–400 MB in all cases, substantially smaller than the footprint of the aligners themselves, which must keep a copy of the reference genome index in memory. For SNAP, the peak memory footprint increased by less than 1.15%. For BWA-MEM, the increase was always less than 5% and for Bowtie 2 less than 13%.

**Table 4:** Overhead of the Qtip tool

			Time (minutes)			Peak memory (gigabytes)		
			Time	+Qtip	% inc	Memory	+Qtip	% inc
ERR050082	Unpaired	Bowtie 2	23.58	25.20	6.89	3.26	3.52	8.25
		BWA-MEM	22.18	23.75	7.08	7.53	7.79	3.40
		SNAP	12.13	13.75	13.32	29.26	29.51	0.85
	Paired	Bowtie 2	57.52	61.35	6.67	3.27	3.66	12.02
		BWA-MEM	57.93	63.38	9.41	7.87	8.26	4.87
		SNAP	11.28	14.42	27.69	30.21	30.55	1.14
ERR050083	Unpaired	Bowtie 2	23.02	24.75	7.55	3.26	3.53	8.28
		BWA-MEM	24.60	26.08	6.03	7.75	8.01	3.33
		SNAP	12.23	13.73	12.31	29.26	29.51	0.85
	Paired	Bowtie 2	63.60	67.40	6.00	3.27	3.66	11.96
		BWA-MEM	61.58	67.52	9.62	7.92	8.31	4.83
		SNAP	11.95	14.68	22.86	30.21	30.55	1.14

This is measured as the increase in running time (left) and peak memory footprint (right) from when the aligner runs by itself (Time) to when the aligner runs in combination with Qtip (+Qtip). % inc columns give the percentage increase. Times are in minutes and memory footprints are in gigabytes

## Methods

### Tandem simulation

**Fig. 3:**
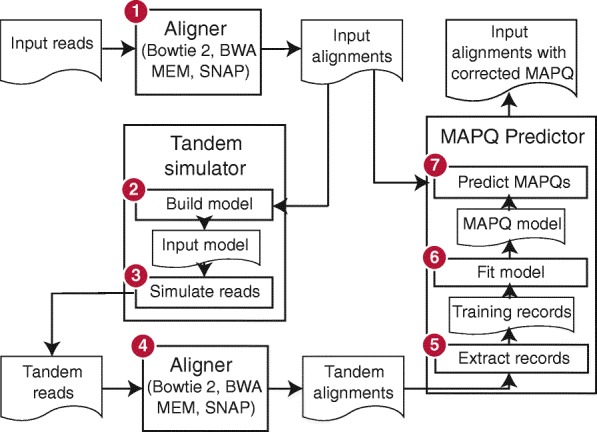
Stages of the Qtip pipeline. Computational steps and intermediate results in Qtip. Numbers denote ordering of steps and arrows denote the flow of data. The input (*upper left*) is a collection of sequencing reads and the ultimate output (*upper right*) is a SAM file containing alignments, where each aligned read’s MAPQ field is set according to Qtip’s prediction

The user specifies a collection of input reads (*R*=*r*_0_,*r*_1_,…,*r*_*n*−1_), a read aligner, alignment parameters, a reference genome in FASTA format, and any other files required, such as a genome index. The tandem simulation framework (Fig. 3) aligns the input reads to the reference genome and predicts a mapping quality *q*_*i*_ for each aligned read. In step 1, input reads are aligned to the reference genome using the specified aligner and parameters. In step 2, the SAM-formatted [3] alignments are parsed and an *input model*, capturing information about the input reads and their alignments, is built. In step 3, the input model and reference genome are used to simulate a new set of reads, called *tandem* reads since they originate from tandem simulation. Each tandem read is from a random location in the genome and is labeled with its true point of origin. In step 4, tandem reads are aligned to the reference genome using the same aligner and parameters as in step 1. In step 5, the alignments produced in step 4 are parsed and converted to *training records*. Because the true point of origin is known, each training record can be labeled as correct or incorrect. In step 6, a model is trained on the records from step 5. In step 7, SAM alignments from step 1 are parsed. For each aligned read, a *test record*, like the training record from step 5, is constructed. Based on the test record, the trained model is applied to predict *q*_*i*_. The alignment’s SAM record is then rewritten substituting *q*_*i*_ in the MAPQ field. New predictions for all input alignments are written in this way.

Importantly, the mapping-quality model trained in step 6 is tailored to the alignment scenario at hand. The aligner and parameters from step 1 are reused in step 4, and tandem reads generated in step 3 mimic the input reads.

To work with the tandem simulation framework, the aligner must report feature data – how well the read aligned, what other alignments were found, what heuristics were used, etc. – used to train and apply the model. This requires modifications to the alignment software. The modifications are not complex and do not affect the efficiency or accuracy of the aligner. However, making appropriate modifications requires knowledge of how the aligner works and of which intermediate alignment results constitute informative features. For this study, we adapted three tools: Bowtie 2 v2.3.2, BWA-MEM v0.7.15, and SNAP v1.0beta.18. Additional file 1: Note 6 provides links to our modifications and details about the modifications made and how features were chosen.

We chose these three aligners both because of their popularity and because they together support a breadth of alignment scenarios. For example, Bowtie 2 and BWA-MEM support local alignment, Bowtie 2 and SNAP support end-to-end alignment, and all three tools support both unpaired and paired-end alignment. Also, all three tools produce their own mapping-quality predictions.

### Read and alignment categories

When predicting mapping quality, Qtip uses a different model depending on whether the alignment is unpaired (*unp*), paired-end and concordantly aligned (*conc*), paired-end and discordantly aligned (*disc*), or paired-end with the opposite end having failed to align (*bad-end*). Qtip trains each model with alignments of the same category. Qtip parameters control the minimum number of tandem reads or pairs of each category to generate. The default number for each category is $45 \sqrt {x}$, where *x* is the number of input alignments of that category. Both the scaling factor and the function are configurable via Qtip’s –sim-function and –sim-factor parameters. Qtip enforces a minimum of 30,000 tandem reads for the *conc* and *unp* categories and 10,000 for the *disc* and *bad-end* categories. The formula for the number of training records is discussed further in Additional file 1: Note 10, with alternatives explored in Additional file 1: Figure S3.

### Input model and simulation of tandem reads

The input model built in step 2 of Qtip (Fig. 3) captures information about the input reads and alignments. Qtip uses this to simulate new tandem reads that are from random genomic locations but are like the input reads in key ways, mimicking their read length distribution, quality strings, and patterns of gaps and mismatches. Tandem paired-end reads additionally mimic the input’s fragment length distribution and relative orientation of the two ends.

To accomplish this, Qtip takes the following approach. For each aligned unpaired read, a *template* data record is created. The template consists of the strand aligned to, the read’s quality string, and the pattern of mismatches and gaps in the alignment as defined by the CIGAR and MD:Z SAM fields. For each aligned pair, the template additionally stores the pair’s inferred fragment length and a flag indicating which end aligned upstream with respect to the genome. Since templates for large datasets can quickly exhaust memory, Qtip uses reservoir sampling to keep a configurable-sized subsample of the templates. The default sample size is 10,000.

In step 3, Qtip uses the input model to simulate tandem reads. To simulate an unpaired tandem read, Qtip randomly draws an unpaired template, with replacement and uniform probability, from those collected in step 2. A new read is constructed from the template by (a) drawing an appropriate-length substring from the reference genome uniformly at random, (b) possibly reverse-complementing it, according to the template strand, (c) mutating the extracted sequence according to the template pattern of mismatches and gaps, and (d) setting the new read’s quality string equal to the template’s. The simulated read’s point of origin is encoded in the read name, allowing later steps to check whether an alignment is correct. The process for simulating a paired tandem read is similar, with fragment length determined by the template. More details are given in Additional file 1: Note 11.

Importantly, some aspects of the input data are hard to mimic. For example, errors made by 454 and Ion Torrent sequencing technologies can manifest as spurious extensions or retractions of homopolymers. Since genome substrings are matched with templates randomly, homopolymer errors in the template will often fail to line up with homopolymers in the substring. Other aspects of the input data are not as difficult to mimic, but happen not to be captured in Qtip’s simulation. For example, if a dataset is enriched or depleted for reads drawn from a particular genomic feature (e.g., coding regions), Qtip’s simulation, which draws reads uniformly at random from across the genome, will not exhibit that pattern. While we demonstrate Qtip performs well despite these deficiencies, they nonetheless illustrate that it is difficult to construct tandem reads that truly mimic input reads in all ways. We return to this in the “Discussion”.

### Mapping-quality model

Given training records derived from tandem reads aligned in step 4, we train a model in steps 5 and 6 that is later used to predict *q*’s for the input alignments. Qtip trains separate models for each alignment category: *unp*, *conc*, *disc*, and *bad-end*. The particular features used to train a model vary depending on the alignment category and read aligner. We briefly summarize these here, but more details are provided in Additional file 1: Note 6.

These features are included regardless of aligner or alignment category: (a) the alignment score of the best alignment, (b) the difference between the alignment score of the best alignment and that of the second-best alignment if one was found, (c) the length of the aligned read, (d) the sum of the base qualities of the aligned bases, and (e) the sum of the base qualities of the soft-clipped bases. For a concordantly aligned pair, the inferred fragment length (from the SAM TLEN field) is also included as a feature.

By default, Qtip uses an implementation of random forests [26] from the scikit-learn [27] library to model and predict mapping qualities. The random forest consists of many decision trees, each trained on a bootstrap sample of the training (tandem) data. Each tree contributes a vote on the probability for whether the given alignment is correct, and the final prediction is the average of the votes. This model is invariant under scaling transformations of features. Training is also efficient, which is important since models are tailored to the scenario at hand, and must be rebuilt anew each time Qtip runs. Finally, it is capable of reporting feature importances, which we examine in more detail in the context of our simulation experiments (Additional file 1: Note 12 and Figures S4–S9). Further details on the model are in Additional file 1: Note 13.

## Discussion

Qtip’s predictions are accurate in various scenarios: various read lengths, unpaired or paired reads, various alignment tools and parameters, etc. We defined novel measures (RCA and RCE) and plots (CID and CSED) for evaluating and plotting mapping-quality predictions. The framework is easy to adapt to other aligners; the aligner must be modified to output feature data in an extra SAM field. Nor is it difficult to add new features to an already-adapted read aligner. Since Qtip’s ensemble tree model is scale-agnostic, scaling guesswork it not necessary when adding a feature.

This framework is also applicable to specialized alignment settings, such as spliced RNA-seq alignment. In that case, a nuanced notion of correctness is needed; we care not only where an alignment lands on the reference but also whether it includes the correct splice junctions. There is room for improvement in predicting mapping qualities for spliced alignments. Popular tools use simplistic prediction functions drawing quality values from a small range of possibilities. TopHat [28] and STAR [29] report a mapping quality of either 0 or 255 (repetitive versus unique) depending on the number of alignments found. Qtip’s approach would produce a full spectrum of values, potentially with large downstream benefits.

Tandem simulation works to the degree that tandem reads can be sampled from the same distribution as input reads. In reality, sampling from the same distribution is not possible. Qtip mimics some aspects of the input data but not others. Homopolymer extensions and retractions are not captured, for example, creating a fundamental difference between tandem and input reads. A tradeoff exists here: Qtip’s simple model mimics some aspects of the input without sacrificing efficiency, whereas a more complex and less efficient model could improve accuracy by mimicking more aspects. A task for future work is to measure various points in this tradeoff space, and to define measures for characterizing how and to what extent a set of tandem reads differs from the input reads.

A question for future work is whether Qtip’s sampling strategy can be improved. A strategy using importance sampling, for example, might favor tandem reads originating from more difficult-to-predict portions of the sample space. Importance might originate from repetitive elements, or from certain patterns of mismatches and gaps. Together with appropriate weighting during model training, this could achieve comparable accuracy while reducing the number of tandem reads required. It could also reduce the prediction variability we see in experiments involving longer reads and more repetitive genomes.

## Conclusion

We presented the tandem simulation framework and the Qtip software tool implementing the framework. To date, strategies for predicting mapping qualities have either been ad hoc or required the user to prepare training data tailored to the scenario at hand. Qtip runs alongside a read aligner and builds an input model, simulates tandem reads, aligns those using the same aligner and parameters, then uses the trained model to predict mapping qualities. The model and training data are produced automatically and are tailored to the scenario at hand. While Qtip adds an overhead to the read alignment process, it is reasonable, with the time overhead in the 6–28% range and the memory overhead in the 1–10% range. This framework, its improved predictions, and the evaluation performed here should make authors of downstream software tools more confident that mapping qualities can be treated as the probabilities they claim to be, and to integrate those probabilities into their models rather than simply thresholding.

## Endnotes

^1^ For a group of alignments sharing the same *Q*, the penalty is averaged across the group’s elements in *C* and *C* ^′^. That is, if $\hat {a}_{k}, \hat {a}_{k+1}, \ldots, \hat {a}_{l}$ is a maximal stretch of alignments sharing the same quality, then $c_{i} = c_{i-1} + \sum _{j=k}^{l} \text {incorrect}(\hat {a}_{j}) / (l-k+1)$ for *k*≤*i*≤*l*.

^2^ For a group of alignments sharing the same *Q*, the corresponding elements of *E* and *E* ^′^ equal the mean squared error of the group. That is, if $\hat {a}_{k}, \hat {a}_{k+1}, \ldots, \hat {a}_{l}$ is a maximal stretch of alignments sharing the same quality, then $e_{i} = e_{i-1} + \sum _{j=k}^{l} (\text {correct}(\hat {a}_{j}) - \hat {p}_{j})^{2} / (l - k + 1)$ for *k*≤*i*≤*l*.

## Additional file

Additional file 1 Supplementary information. Contains Supplementary Notes 1–13, Figures S1–S9, and Tables S1–S4. (PDF 202 kb)
