# Supplementary material for: A tandem simulation framework for predicting mapping quality
Source: Genome Biol. 2017 Aug 10;18:152. doi: 10.1186/s13059-017-1290-3 (PMC5557537; doi:10.1186/s13059-017-1290-3)
Supplement: Additional file 1 — Supplementary information. Contains Supplementary Notes 1–13, Figures S1–S9, and Tables S1–S4. (PDF 202 kb) [file 13059_2017_1290_MOESM1_ESM.pdf]

Supplementary data for: A tandem simulation framework for predicting mapping quality

Ben Langmead[1,2]

[1]Department of Computer Science, Whiting School of Engineering, Johns Hopkins University

[2]Department of Biostatistics, Bloomberg School of Public Health, JHU

Note 1 Alignment heuristics in Bowtie 2

We elaborate our discussion of alignment heuristics with a few observations about how Bowtie 2 searches for alignments.

1. Only alignments at or above an *alignment score threshold* S_{min} are considered. These are called *valid*.
2. *Local similarity filters* require that one or more substrings of the read to align with few or no differences. For instance, a filter might require that alignments contain a length-k stretch of nucleotides that match the reference exactly. For long enough k, this disqualifies otherwise valid alignments.
3. *Repeat filters* avoid the extensive and mostly unproductive work performed when many candidates pass the local similarity filter. Say we take a 20 nt read then use a fast index lookup to find all reference locations where the first 10 nt of the read occur exactly. Say there are 100,000 such places. Instead of examining each candidate, Bowtie 2 will examine a subset and ignore the rest.
4. *Early stopping* halts alignment work once it seems to have become unproductive. For instance, the aligner might stop searching once a certain number of consecutive index queries have failed to yield a valid alignment.

Note 2 Prevalence of alignment errors

We defined three categories of alignment error:

1. The read is reported to have originated from a locus in the reference genome, but actually originates from a DNA sequence not included in the reference
2. No alignment to the reference is found, but the read actually originates from some locus in the reference
3. An alignment to locus L_r in the reference is reported, but the read actually originates from a different locus in the reference, L_t

To measure prevalence of these errors in a realistic setting, we conducted three sets of simulation experiments. In all three, most simulated reads were derived from a known "target" reference genome, either human (GRCh38) or mouse (GRCm38), with genetic variation and sequencing errors added. These represent reads from a donor individual that have their true point of origin in the reference genome. We also simulated two types of foreign sequences, having their true point of origin outside the reference genome. The first type of foreign sequence consists of species present in laboratories and on humans that have been shown to contaminate sequencing datasets. The second consists of sequence from a CHM1 hydatidiform mole assembly not present in the GRCh38 assembly. These represent sequences that are present in a donor individual but missing from the reference genome. Both types of foreign sequence are important components of Category-1 errors [3]. All reads are aligned to the target reference only. The foreign sequences from the contaminating genomes are included in all experiments; the CHM1 sequences are included in one of the two human experiments, as indicated in Table S1.

To obtain the first kind of foreign sequence, we simulated 7% of the reads from contaminating genomes: 1% each from mouse (GRCm38), the bacteria *Propionibacterium acnes*, *Mycolpasma Hominis*, *Mycolpasma fermentans*, *Acholeplasma laidlawii*, and *Mycolpasma hyorhinis*, and the fungus *Malassezia globosa*. We arrived at these contaminants after sur-

veying studies on sequencing data contamination [5, 11, 13]. When a read from one of these genomes aligns to the GRCh38 primary assembly, we call this a Category-1a error.

For the two experiments that did not include foreign sequence from CHM1, we simulated the remaining 93% of reads from the reference genome. For the third experiment, we included CHM1-specific sequence by simulating 93% of the reads from a concatenation of the GRCh38 reference and a collection of sequences of length at least 260 present in the CHM1 assembly but not in GRCh38. Note 3 gives further detail on how the CHM1-specific sequences were obtained. When a read from the CHM1 assembly aligns to GRCh38, we call this a Category-1b error.

We simulated four samples for each of the three experiments: unpaired and paired-end, 100-nt and 250-nt samples. We ran Mason as described in Note 4. We aligned reads with Bowtie 2 v2.3.2 as described in Note 5. In one experiment we inverted the roles of the mouse and human genomes, using mouse as the target and human as one of the contaminant genomes. For each aligned sample, we counted errors of the three categories and tabulated them as a percentage of the simulated reads and as a percentage of the errors (Table S1).

As expected, longer reads yield fewer errors than shorter reads and paired-end reads yield fewer errors than unpaired reads. A large majority of the errors, ranging from 95.8-99.7%, are of category 3. The next largest, ranging from 0.2-3.9% of errors, are of category 2. These numbers could be reduced using more sensitive alignment parameters. Category 1 errors range from 0.27–0.46% of errors in the more realistic experiment that included CHM1 sequence, and from 0.05–0.014% in the other two experiments. The large fraction of category 3 errors underscores the need for better methods for predicting mapping quality.

Target reference			Category 1a (contamination)		Category 1b (CHM1)		Category 2		Category 3	
			% reads	% errors	% reads	% errors	% reads	% errors	% reads	% errors
GRCh38	100	Unpaired	0.006	0.144	-	-	0.159	3.567	4.287	96.289
		Paired	0.004	0.136	-	-	0.079	2.657	2.888	97.206
	250	Unpaired	0.002	0.100	-	-	0.065	2.713	2.325	97.187
		Paired	0.002	0.094	-	-	0.073	3.902	1.794	96.004
GRCh38+CHM1	100	Unpaired	0.006	0.144	0.009	0.205	0.164	3.692	4.266	95.960
		Paired	0.004	0.136	0.005	0.178	0.078	2.622	2.884	97.064
	250	Unpaired	0.002	0.099	0.009	0.363	0.067	2.758	2.348	96.780
		Paired	0.002	0.093	0.003	0.180	0.073	3.884	1.809	95.843
Mouse	100	Unpaired	0.005	0.064	-	-	0.065	0.822	7.809	99.114
		Paired	0.003	0.056	-	-	0.029	0.502	5.760	99.442
	250	Unpaired	0.003	0.053	-	-	0.012	0.238	4.895	99.709
		Paired	0.002	0.051	-	-	0.013	0.340	3.839	99.609

Table S1: Prevalence of errors of different categories when analyzing Mason-simulated samples using Bowtie 2. The "% reads" column gives percent of all the reads, both correctly and incorrectly aligned, having that error category, and "% errors" gives percent of errors having that error category.

Note 3 Obtaining CHM1-specific sequences

We used MUMmer and nucmer [9] to align the MHAP CHM1 assembly [2] to the GRCh37 primary assembly. We analyzed the resulting alignment using Assemblytics [12], with results available at: `http://assemblytics.com/analysis.php?code=human`. We also downloaded the MHAP CHM1 assembly (accession: GCA_000772585) from Genbank [1].

Similarly, we used MUMmer and nucmer [9] to align the MHAP CHM1 assembly [2] assembly to the GRCh38 primary assembly. We analyzed the resulting alignment using Assemblytics [12], with the results available at: `http://assemblytics.com/analysis.php?code=sIOvqHUKNaCpU8NXdcTS`. This assembly is like the one used above (accession: GCA_000772585), but with low-support contigs (having fewer than 50 supporting reads) removed. We also downloaded this version of the assembly from the University of Maryland at: `http://gembox.cbcb.umd.edu/mhap/asm/human.quiver.ctg.fasta.gz`.

We downloaded the Assemblytics results from both alignments by clicking the "Download zip file of all results" link. Using the CHM1 assemblies and the structural variation BED file reported by Assemblytics, we extracted all insertions in the CHM1 assembly with respect to the reference genome with length at least 260. The minimum length restriction is required by Mason for simulations of 250 nt reads. We included 25 nt of overhang on either side of the extracted CHM1 sequences, to allow for reads aligning to the cusps. We constructed new "+CHM1" versions of the reference genomes by appending CHM1-specific sequences to the FASTA file containing the reference.

Note 4 Simulations

Mason. To generate unpaired samples we ran Mason v0.1.2 [7] with these parameters:

```
mason illumina -hn 2 -i -s (seed) -sq -n (read_len) -N 4000000 \
      -o (output_fastq) (genome_fasta)
```

`illumina` causes Mason to simulate Illumina-like reads. `-hn 2` causes Mason to simulate 2 haplotypes. `-i` causes Mason to include information about the read's true point of origin in the read name, which is useful later for determining which alignments are correct. `-s` sets the pseudo-random seed, which we set arbitrarily for our experiments. `-sq` causes Mason to simulate base qualities. `-n` specifies the read length. `-N` specifies the number of reads to simulate. `-o` specifies the output file.

To generate paired-end samples we ran Mason with these parameters:

```
mason illumina -hn 2 -i -s (seed) -sq -mp -rn 2 -ll (mean) -le (stddev) \
        -n (read_len) -N 4000000 -o (output_fastq) (genome_fasta)
```

`illumina` causes Mason to simulate Illumina-like reads. `-hn 2` causes Mason to simulate 2 haplotypes. `-i` causes Mason to include information about the read's true point of origin in the read name, which is useful later for determining which alignments are correct. `-s` sets the pseudo-random seed, which we set arbitrarily for our experiments. `-sq` causes Mason to simulate base qualities. `-mp` causes Mason to simulate paired-end reads. `-rn 2` enables "one-based slash suffix" read naming. `-ll` and `-le` are discussed below. `-n` specifies the read length. `-N` specifies the number of reads to simulate. `-o` specifies the output file.

For the paired-end samples, we ran Mason with parameters `-ll 3L -le L`. These establish a mean ($3L$) and standard deviation (L) for the fragment length distribution. This means that some simulated fragments will fall outside the range $2L-4L$, despite the fact that this is the range of fragment lengths that the aligners will consider "concordant"

(Note 5). This is desirable; for our paired-end samples, we would like the input to include reads that yield all three alignment categories: *conc*, *disc* and *bad-end*.

Mason v0.1.2 cannot handle very short reference sequences. To work around this, we removed all reference sequences shorter than 10,000 bases at the outset. We filtered out 90 short sequences from the GRCh38 assembly, totaling 229,926 nt. We filtered out one 4,262-nt-long sequence from the GRCh37 assembly. We filtered out one 1,976-nt-long sequence from the GRCm38 assembly. And we filtered out 5 short sequences from the AGPv4 assembly, totaling 37,762 nt.

When simulating paired-end reads, Mason v0.1.2 would sometimes generate pairs where the end that one would expect to find upstream would actually be found downstream. For example, in a typical Illumina paired-end protocols, the two ends of a fragment originating from the forward reference strand would align with (a) end 1 upstream of end 2, (b) end 1 aligning in the forward orientation, and (c) end 2 aligning in the reverse orientation. But for around 0.10–0.15% of the pairs in a Mason-simulated sample, we found that the upstream/downstream relationship between the ends was reversed. These were filtered out prior to our experiments.

wgsim. wgsim is one of the simulators we evaluate in Note 8. To generate unpaired samples with wgsim, we ran it with these parameters:

```
wgsim -S (seed) -1 (read_len) -2 (read_len) -N 4000000 \
        (genome_fasta) (output_fastq)
```

`-S` sets the pseudo-random seed, which we set arbitrarily for our experiments. `-1` and `-2` specify the read lengths for either end of a paired-end read. `-N` specifies the number of reads to simulate. The last two parameters specify the reference genome FASTA file and the output FASTQ file.

To generate paired-end samples we ran wgsim with these parameters:

```
wgsim -S (seed) -d (mean) -s (stddev) -1 (read_len) -2 (read_len) \
```

```
        -N 4000000 (genome_fasta) (output_fastq_1) (output_fastq_2)
```

-S sets the pseudo-random seed, which we set arbitrarily for our experiments. -d sets the mean fragment length and -s sets the fragment length standard deviation. -1 and -2 specify the read lengths for either end of a paired-end read. -N specifies the number of reads to simulate. The last two parameters specify the reference genome FASTA file and the output FASTQ files.

Art. Art is one of the simulators we evaluate in Note 8. To generate unpaired samples with Art, we ran it with these parameters:

```
art_illumina -sam -na -rs (seed) -l (read_len) -f (fold_coverage) \
        -i (genome_fasta) -o (output_fastq)
```

-sam enables SAM output. Information about a simulated read's point of origin is encoded in the SAM file. -na suppresses the ALN alignment file. -rs sets the pseudo-random seed, which we set arbitrarily for our experiments. -l specifies the read length. -f specifies the fold coverage. We set this to be high enough that we are eventually able to obtain 4 million reads or pairs per sample. -i specifies the reference genome FASTA file. -o specifies the output file.

To generate paired-end samples we ran Art with these parameters:

```
art_illumina -sam -na -p -rs (seed) -m (mean) -s (stddev) -l (read_len) \
        -f (fold_coverage) -i (genome_fasta) -o (output_fastq)
```

-sam enables SAM output. Information about a simulated read's point of origin is encoded in the SAM file. -na suppresses the ALN alignment file. -rs sets the pseudo-random seed, which we set arbitrarily for our experiments. -m specifies the mean fragment length. -s specifies the standard deviation. -l specifies the read length. -f specifies the fold coverage. We set this to be high enough that we are eventually able to obtain 4 million reads or pairs per sample. -i specifies the reference genome FASTA file. -o specifies the output file.

Note 5 Read alignment

Bowtie 2. For paired-end samples, we ran Bowtie 2 with the `-I` and `-X` parameters to set the minimum and maximum concordant fragment lengths. We specified `-I 2L -X 4L` where `L` corresponds to the `-ll` and `-le` parameters specified when running Mason. For experiments where we varied sensitivity level, we also specified a sensitivity parameter: `--very-fast`, `--fast`, `--sensitive` or `--very-sensitive`. For experiments where Bowtie 2 was run in local alignment mode, we also specified the `--local` parameter. Additionally, Qtip always specifies the `--mapq-extra` option when running Bowtie 2, which enables feature-reporting features. When running Qtip together with Bowtie 2, the user may also specify additional Bowtie 2 parameters on the command line.

BWA-MEM. For paired-end samples, we ran BWA-MEM with the `-I` option to set the minimum and maximum concordant fragment lengths. BWA-MEM's default behavior, unlike either Bowtie 2 or SNAP, is to infer the fragment length distribution over a sample of the input reads. By specifying `-I` we disabled that automatic inference. This was to ensure that the read aligners worked similarly and, in particular, that all agreed on which fragment lengths should be considered concordant. The `-I` parameter sets the mean and standard deviation of the fragment length distribution. BWA-MEM consider any fragment length within 4 standard deviations of the mean to be concordant. Thus, we set `-I 3L,L/4`, achieving the same result as we did above with Bowtie 2's `-I` and `-X` parameters. When running Qtip together with BWA-MEM , the user may also specify additional BWA-MEM parameters on the command line.

SNAP. For paired-end samples, we ran SNAP with the `-s` parameter to set the minimum and maximum concordant fragment lengths. We specified `-s L 3L` indicating that the distance between the leftmost base of the upstream made and the leftmost base of the downstream made is allowed to vary from L to $3L$, achieving the same result as we did

above with Bowtie 2's `-I` and `-X` parameters. Additionally, Qtip always sets SNAP's `-=` parameter for reasons described in Note 11. When running Qtip together with SNAP, the user may also specify additional SNAP parameters on the command line.

Note 6 Modifications to read aligners

We modified three alignment tools — Bowtie 2, BWA-MEM , and SNAP— to output feature data needed by Qtip. To make the modifications, we studied each tool's source code and identified data that could be relevant to the mapping quality prediction task. We started by studying how the tool itself predicts mapping quality, since the inputs to those routines are candidate features. In all three cases, we added more features beyond these, usually with the goal of capturing more information about the repetitiveness of the read and how many of its "seeds" either failed to align or aligned repetitively.

Here we examine each tool, discussing (a) how the tool predicts mapping quality, (b) which features we re-used from that process, and (c) which additional features we added.

Bowtie 2 Bowtie 2's mapping quality calculation uses a lookup table, with the lookup keys being functions of (a) the best alignment's score, (b) the second-best alignment's score, (c) whether end-to-end or local alignment were used, (d) whether the read aligned concordantly in a pair. We preserve (a) and (b) in the features we elect to report from Bowtie 2. We do not need to report either (c) or (d) because these follow naturally from how Qtip works. In the case of (c), the training data is tailored to match the alignment parameters used to align the input data; the training data will always match the input data in terms of the alignment mode used. In the case of (d), Qtip trains separate models for separate alignment categories (*conc*, *disc*, *unp*, *bad-end*), and always uses the appropriate model for prediction.

Following is a list of the unpaired features output in the modified version of Bowtie 2:

- Best alignment score
- Difference between the best and second-best alignment score, or NA if no second-best alignment was found
- Difference between the best and second-best edit distance, or NA if no second-best alignment was found

- Number of seeds for which Bowtie 2 sought an alignment (*attempted* seeds) divided by the number of nucleotides in the read
- Fraction of attempted seeds that aligned uniquely (to exactly one place in the reference)
- Same as previous but limited to seeds that aligned in the same orientation as the overall reported alignment
- Fraction of attempted seeds that aligned repetitively (to more than one place in the reference)
- Same as previous but limited to seeds that aligned in the same orientation as the overall reported alignment
- Mean number of alignments found for any attempted seed
- Same as previous but limited to seeds that aligned in the same orientation as the overall reported alignment

Following is a list of the paired-end features output in the modified version of Bowtie 2:

- Sum of the alignment scores for both ends of the best paired-end alignment
- Difference between the best and second-best paired-end alignment score (i.e. the sum of the scores of the two ends), or NA if no second-best alignment was found
- Difference between the best and second-best paired-end edit distance (i.e. the sum of the edit distances of the two ends), or NA if no second-best alignment was found

Note 12 discusses the relative importance of these features as we measured in our simulation experiments.

BWA-MEM. BWA-MEM 's mapping quality calculation depends on, among other factors, (a) the best alignment's score, (b) the second-best alignment's score, (c) the number of alignments "tied" for second-best, (d) the number of aligned bases (i.e. excluding any

soft-clipped bases), (e) the percent identity, summarized across the aligned bases, (f) the *seed coverage*, which roughly equals the mean number of times a read base is covered by an aligned seed. We use these features and others in Qtip.

Following is a list of the unpaired features output in the modified version of BWA-MEM :

- Best alignment score
- Edit distance of the best alignment
- Difference between the best and second-best alignment score, or NA if no second-best alignment was found
- Number of alignments “tied” for second-best
- Fraction of seeds that aligned repetitively
- Seed coverage

Following are the paired-end features output in the modified version of BWA-MEM :

- Difference between the best and second-best paired-end alignment score (i.e. the sum of the scores of the two ends), or NA if no second-best alignment was found
- Sum of alignment scores of the two ends of the best paired-end alignment
- Number of paired-end alignments “tied” for second-best
- Fraction of seeds from either end of the pair that aligned repetitively
- Seed coverage over both ends of the pair

Note 12 discusses the relative importance of these features as we measured in our simulation experiments.

SNAP. For a given read, SNAP assigns a probability to each alignment found for that read. SNAP’s procedure for calculating the best alignment’s mapping quality mostly consists of dividing the best alignment’s probability by the sum of the probabilities of all the other alignments found. The probability of a given alignment is function of the

length of the read, the read's quality values, and a collection of parameters giving the prior probability of a variant, gap opening, gap extension, etc. We use these probabilities and other features in Qtip.

Following are the unpaired features we output in the modified version of SNAP:

- Edit distance of the best alignment
- Difference between best and second-best alignment's edit distance, or NA if no second-best alignment was found
- Probability of the best alignment divided by the total probability of all the alignments found
- Number of seeds ignored because they matched in the genome too many times
- Minimum number of times a matching seed matched in the genome
- Number of seeds that failed to match the genome
- Mean number of matches per seed

Following are the paired-end features we output in the modified version of SNAP:

- Difference between the best and second-best paired-end edit distance (i.e. the sum of the scores of the two ends), or NA if no second-best alignment was found
- Total number of seeds in the pair ignored because they matched in the genome too many times
- Minimum number of times a matching seed matched in the genome across both ends of the pair
- Number of seeds that failed to match the genome across both ends of the pair
- Mean number of matches per seed across all seeds in the pair

Note 12 discusses the relative importance of these features as we measured in our simulation experiments.

Qtip parses feature data from the extra `ZT:Z` SAM field. So once we identified a feature, we enable Qtip to use that feature simply by printing it as an additional value in

the `ZT:Z` field. This field contains comma-separated numeric data. The following is an example of an alignment produced by BWA-MEM with the `ZT:Z` field. The `ZT:Z` field is not printed for unaligned reads, and Qtip will ignore these.

Qtip also ignores the small number of chimeric alignments reported by BWA-MEM. Proper handling of chimeric alignments is future work.

In addition to the features discussed above, which are reported by the read aligners in the `ZT:Z` field, Qtip adds a few additional features, specifically:

- Length of the read or end
- Sum of the base qualities of the aligned bases in the read or end
- Sum of the base qualities of the soft-clipped bases in the read or end

Finally, for a given end of a paired-end alignment, whether concordant or discordant, feature data for the opposite end is included in the record for the given end. I.e., in the record for a given end of a paired-end read, you will find aligner-specific unpaired feature data, aligner-specific paired-end feature data, generic features, and both aligner-specific and generic features for the opposite end.

When the training and test data are compiled within Qtip, columns in which all elements are equal are eliminated. E.g., if the input consists of reads all of the same length, then the read length will be identical for all records and the corresponding column will be dropped from the training and test matrices. Similarly, if the input consists of reads aligned in end-to-end alignment mode (Bowtie 2's default), then the sum of the base qualities of the soft-clipped bases will always be 0, and the column corresponding to that feature will again be dropped.

Availability of aligner modifications. Bowtie 2 v2.3.2 has the appropriate feature-printing code built in; earlier versions of Bowtie 2 do not have this code. The `--mapq-extra` parameter instructs Bowtie 2 v2.3.2 to print feature data in the `ZT:Z` extra SAM field. To run this version of Bowtie 2 in combination with Qtip, use Qtip's `--bt2-exe` parameter.

Qtip comes with a source-code patch that causes BWA-MEM to output feature data for use with Qtip. To create a modified version of BWA-MEM , download the BWA-MEM v0.7.15 source archive from `https://sourceforge.net/projects/bio-bwa/files/`. Once extracted, change to the BWA-MEM v0.7.15 source directory and run:

```
patch -p1 < /path/to/qtip/software/bwa/bwa_conc_flags_0.7.15.patch
```

Then build the binaries as usual. To run this version of BWA-MEM in combination with Qtip, use Qtip's `--bwa-exe` parameter. The modified BWA-MEM will print the feature data in the `ZT:Z` extra SAM field.

Qtip comes with a source-code patch that causes SNAP to output feature data for use with Qtip. To create a modified version of SNAP, download the SNAP v1.0beta.18 source archive from `http://snap.cs.berkeley.edu`. Once extracted, change to the SNAP v1.0beta.18 source directory and run:

```
patch -p1 < /path/to/qtip/software/snap/snap_features_patch.diff
```

Then build the binaries as usual. To run this version of SNAP in combination with Qtip, use Qtip's `--snap-exe` parameter. The modified SNAP will print the feature data in the `ZT:Z` extra SAM field.

			100 nt				250 nt			
			RCA		RCE		RCA		RCE	
			mean	sd	mean	sd	mean	sd	mean	sd
Unpaired	Bowtie 2	GRCh37	-11.22	1.08	-24.43	0.66	-15.02	0.39	-28.37	0.38
		GRCh37+CHM1	-10.77	0.71	-24.22	0.35	-12.78	2.07	-27.67	1.14
		GRCh38	-7.43	1.82	-18.53	2.61	-16.82	2.01	-19.97	0.44
		GRCh38+CHM1	-7.39	1.31	-18.50	1.26	-16.36	2.03	-19.76	1.07
	BWA-MEM	GRCh37	-14.49	2.29	-49.54	0.43	-9.14	2.09	-52.31	0.38
		GRCh37+CHM1	-15.55	0.81	-49.34	0.29	-11.62	6.27	-51.42	0.35
		GRCh38	-15.49	0.58	-47.42	0.37	-15.78	0.57	-51.14	0.31
		GRCh38+CHM1	-15.53	0.56	-47.14	0.33	-15.57	0.79	-50.80	0.54
	SNAP	GRCh37	-15.94	0.32	-36.86	0.23	-9.53	3.88	-28.57	0.47
		GRCh37+CHM1	-16.39	0.93	-36.95	0.29	-6.07	6.84	-27.66	0.44
		GRCh38	-19.58	0.18	-36.58	0.47	-14.74	0.27	-25.47	0.32
		GRCh38+CHM1	-19.51	0.24	-36.69	0.37	-14.75	0.32	-25.29	0.71
Paired	Bowtie 2	GRCh37	-48.62	0.23	-65.07	0.25	-54.09	1.55	-68.39	0.31
		GRCh37+CHM1	-46.72	0.59	-64.70	0.14	-53.18	1.36	-68.06	0.32
		GRCh38	-34.93	1.00	-54.32	0.19	-47.93	0.26	-57.77	0.19
		GRCh38+CHM1	-34.92	0.93	-54.28	0.21	-47.07	0.35	-57.54	0.19
	BWA-MEM	GRCh37	-13.33	0.23	-45.70	0.27	-10.08	0.75	-47.58	0.31
		GRCh37+CHM1	-14.21	0.70	-45.02	0.39	-15.31	1.41	-46.90	0.36
		GRCh38	-14.19	0.16	-41.35	0.19	-12.36	0.46	-42.78	0.29
		GRCh38+CHM1	-13.89	0.23	-40.97	0.25	-12.68	0.33	-42.52	0.27
	SNAP	GRCh37	-56.53	1.99	1.39	2.34	-42.89	7.63	13.17	3.95
		GRCh37+CHM1	-54.39	1.67	1.79	1.73	-42.30	8.09	14.39	5.07
		GRCh38	-51.36	0.98	-11.16	1.12	-51.32	1.45	4.34	2.29
		GRCh38+CHM1	-51.11	0.97	-11.43	0.86	-50.91	1.48	3.88	2.06

Table S2: Relative change in area under CID (RCA) and relative change in sum of squared error (RCE) for various aligners and two versions of the human reference genome, expressed as percent change. Some experiments simulated reads from just the reference (“GRCh37,” “GRCh38”) while other simulated from insertions in the CHM1 assembly relative to the reference assembly (“GRCh37+CHM1,” “GRCh38+CHM1”). The experiments used 100 nt or 250 nt reads, and unpaired or paired-end reads, as indicated. Results are means and standard deviations over 10 random trials, repeated starting from the input modeling step.

Note 7 Varying aligner sensitivity

We used Qtip together with Bowtie 2 to align and predict mapping qualities for the Mason-simulated 100 nt and 250 nt unpaired and paired-end samples. We repeated each experiment four times, varying Bowtie 2's sensitivity parameter: `--very-fast`, `--fast`, `--sensitive` or `--very-sensitive`. We also tried Qtip both in its (default) end-to-end alignment mode and in its local alignment mode. We calculated RCA and RCE for all 10 trials of all experiments (Table S3) and plotted CSED for the first trial of the experiments that used the 100 nt reads (Figure S1).

Qtip-predicted mapping qualities are superior overall to Bowtie 2's in every scenario tested, as indicated by the negative RCAs and RCEs (Table S3). Variability of RCAs and RCEs across trials is generally modest, but higher for `--very-sensitive` mode. Standard deviations range up to 5.03.

CSED curves (Figure S1) again show that for some ranges of cutoffs, aligner-reported mapping qualities are better at minimizing cumulative squared error, corresponding to the portions of the CID curves that rise above $y = 0$. This is most clearly observed for unpaired `--very-sensitive` alignment. But Qtip provides superior predictions for the vast majority of cutoffs, including nearly all cutoffs for the paired-end experiments.

			End-to-end				Local			
			RCA		RCE		RCA		RCE	
	Length	Sensitivity	mean	sd	mean	sd	mean	sd	mean	sd
Unpaired	100	–very-fast	-9.55	1.04	-20.73	1.13	-17.06	0.37	-47.04	0.23
		–fast	-8.33	0.28	-19.96	0.26	-13.66	0.37	-36.52	0.42
		–sensitive	-7.51	1.47	-18.74	1.57	-11.58	0.66	-29.62	0.45
		–very-sensitive	-6.53	1.65	-18.32	2.62	-9.01	1.78	-21.75	4.61
	250	–very-fast	-25.26	0.77	-32.32	0.43	-32.44	1.59	-52.37	0.40
		–fast	-18.75	1.63	-24.21	0.71	-24.16	1.40	-35.51	0.60
		–sensitive	-16.38	2.07	-19.25	1.80	-22.27	3.55	-29.07	1.12
		–very-sensitive	-16.10	2.64	-17.58	2.40	-15.59	5.03	-20.87	1.89
Paired	100	–very-fast	-32.56	1.00	-50.48	0.26	-67.65	0.21	-49.77	0.63
		–fast	-33.69	0.51	-51.75	0.22	-66.36	0.12	-51.48	0.51
		–sensitive	-35.56	0.65	-54.34	0.12	-63.63	0.33	-52.06	0.36
		–very-sensitive	-37.00	0.32	-56.43	0.09	-60.54	0.30	-53.97	0.63
	250	–very-fast	-46.44	0.45	-57.22	0.25	-71.51	0.60	-62.20	1.86
		–fast	-45.04	3.03	-58.18	0.19	-69.35	0.34	-63.67	1.36
		–sensitive	-47.42	0.72	-57.80	0.14	-67.00	1.35	-61.53	2.56
		–very-sensitive	-46.76	1.70	-57.93	0.22	-63.41	0.55	-62.27	1.48

Table S3: Relative change in area under CID (RCA) and relative change in sum of squared error (RCE) when running Qtip and Bowtie 2 at various levels of sensitivity on Mason-simulated Illumina-like samples. Relative change is expressed as a percent. Each simulated sample consists of 4 million reads or pairs. Bowtie 2 is run in either end-to-end or local alignment mode as indicated. Results are means and standard deviations over 10 random trials, repeated starting from the input modeling step.

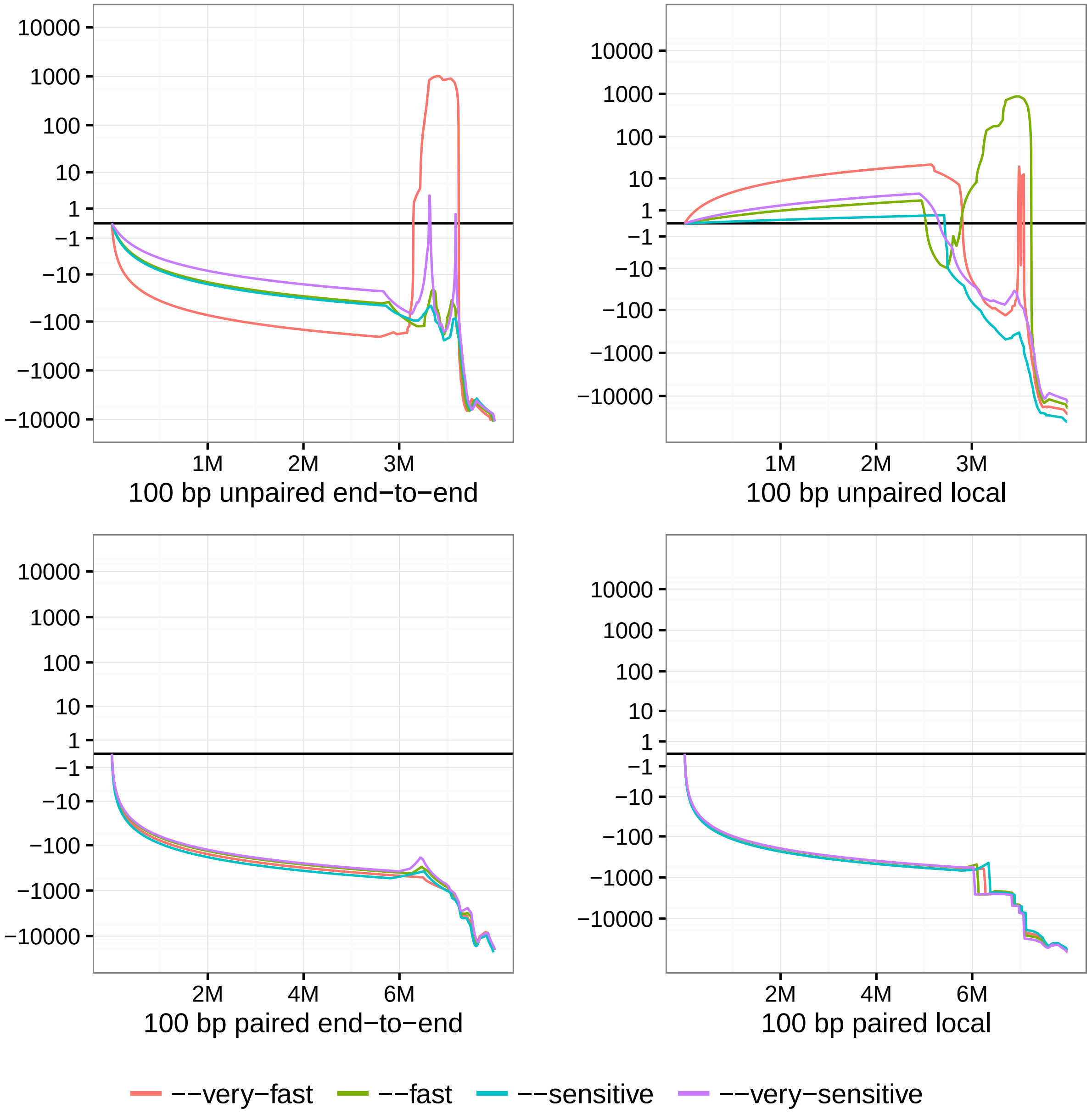

Figure S1: Cumulative squared-error difference (CSED) plot from running Qtip and Bowtie 2 and varying Bowtie 2's sensitivity level. The input reads are Mason-simulated Illumina-like 100 nt samples, each consisting of 4 million reads/pairs. The horizontal axis indicates cumulative number of reads/ends passing the cutoff, with the left-hand extreme corresponding to a high mapping-quality cutoff and right-hand extreme corresponding to a low cutoff. Results for unpaired samples are shown on top, and paired on bottom. Bowtie 2 is run in its (default) end-to-end mode in the case of the left-hand plots, and in local mode in the case of the right-hand plots.

Note 8 Varying read simulator

Different read simulators make different decisions about how to emulate the sequencing process and its errors and biases. To study how this impacts Qtip's predictions, we ran a series of experiments using Mason [7] and the popular wgsim (`https://github.com/lh3/wgsim`) and Art [8] simulators. For all three, we simulated 100 nt and 250 nt samples, both unpaired and paired-end. See Note 4 for the commands used and details about simulated fragment lengths. We then used Qtip together with Bowtie 2 to align and predict mapping qualities. We calculated RCA and RCE for all 10 trials of all experiments (Table S4) and plotted CID for the first trial (Figure S2). Overall, the results show the choice of simulator does not have a large effect on the outcome of the experiment. Differences in prediction performance on the paired-end samples may be due in part to the different fragment length distributions generated by the simulators, as discussed in Note 4.

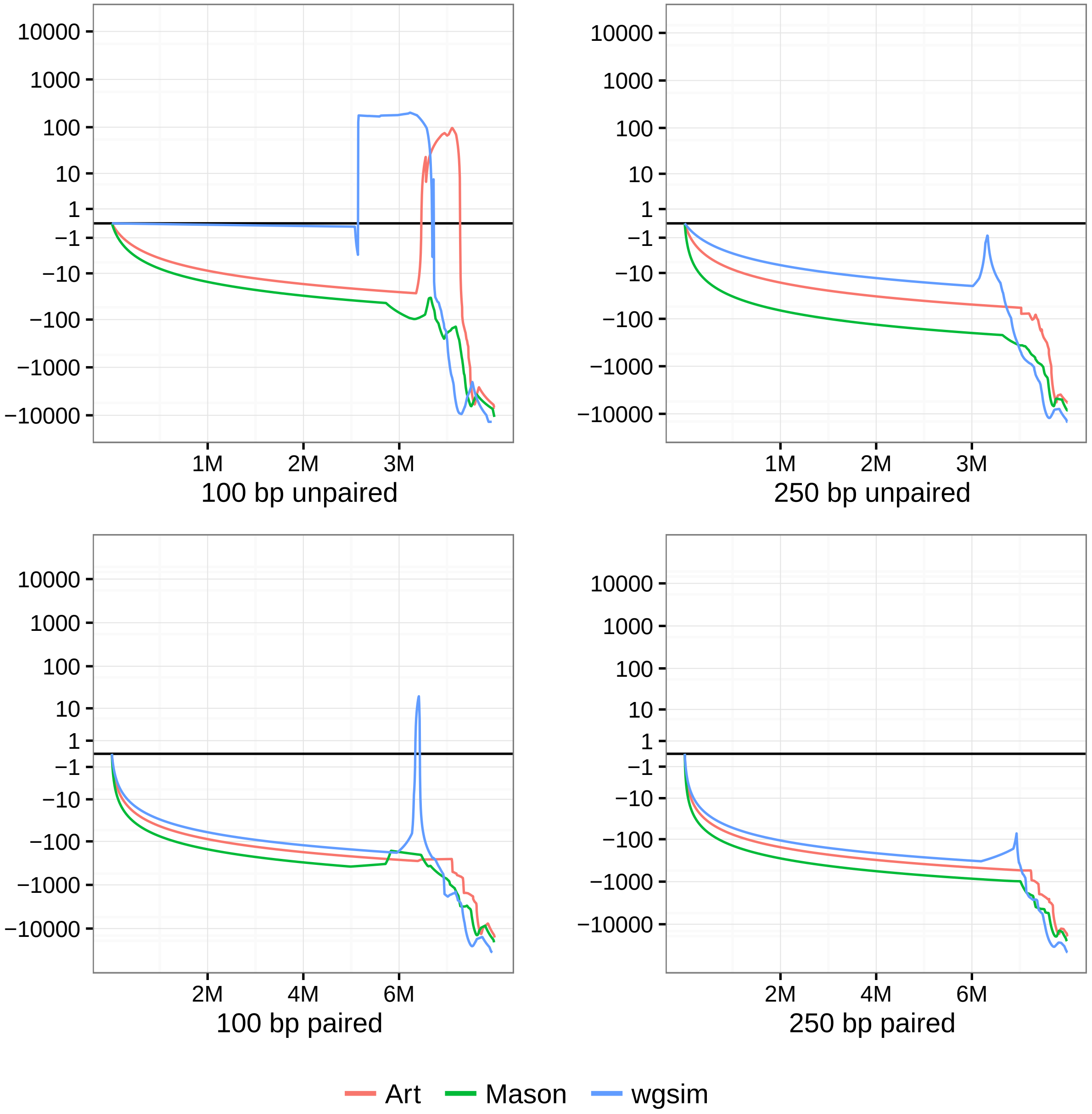

Figure S2: Cumulative squared-error difference (CSED) plot from running Qtip and Bowtie 2 on samples simulated from three different read simulators: Mason, wgsim and Art. Each sample consists of 4 million reads or pairs. The horizontal axis indicates cumulative number of reads/ends passing the cutoff, with the left-hand extreme corresponding to a high quality cutoff and right-hand extreme to a low cutoff.

			RCA		RCE	
			mean	sd	mean	sd
Unpaired	100 nt	Art	-6.26	0.35	-15.87	0.66
		Mason	-7.43	1.82	-18.53	2.61
		wgsim	-9.06	0.25	-19.60	0.90
	250 nt	Art	-12.41	0.84	-19.83	0.53
		Mason	-16.82	2.01	-19.97	0.44
		wgsim	-18.52	0.32	-26.93	0.50
Paired	100 nt	Art	-26.02	0.83	-41.98	0.26
		Mason	-34.93	1.00	-54.32	0.19
		wgsim	-26.37	0.84	-46.00	0.19
	250 nt	Art	-40.37	0.36	-47.21	0.30
		Mason	-47.93	0.26	-57.77	0.19
		wgsim	-39.18	1.10	-54.41	0.21

Table S4: Relative change in area under CID (RCA) and relative change in sum of squared error (RCE) for samples simulated by various simulators. Bowtie 2 end-to-end alignment was used in all cases. Relative change is expressed as a percent. The experiments used 100 nt or 250 nt reads, and unpaired or paired-end reads, as indicated. Results are means and standard deviations over 10 random trials, repeated starting from the input modeling step.

Note 9 Variant calling experiment

Alignment: We used Qtip v1.6.2 together with Bowtie 2 v2.3.2 to align the paired-end sequencing reads from ERR194147. The Qtip command used was:

```
qtip \
    --ref ${FASTA_PATH}/hg38.fa \
    --m1 ERR194147_1.fastq --m2 ERR194147_2.fastq \
    --index ${INDEX_PATH}/hg38.fa \
    --bt2-exe ${BOWTIE2_PATH}/bowtie2 \
    --keep-intermediates \
    --output-directory ${ODIR} \
    --write-orig-mapq \
    --write-precise-mapq \
    --temp-directory ${TEMP} \
    -- ${BOWTIE2_ARGS}
```

The Bowtie 2 arguments used (inserted where `${BOWTIE2_ARGS}` appears above) are: `-I 0 -X 550 -t -p 24`. The first two parameters set minimum and maximum fragment length to 0 and 550 respectively. The `-t` parameter enables extra timing output. The `-p 24 --reorder` parameters run Bowtie 2 with 24 threads while keeping output alignments in an order corresponding to the input reads.

With these parameters, the output directory from Qtip contains both an `input.sam` file with the original mapping qualities, and a `final.sam` file with the Qtip-predicted mapping qualities for the same alignments.

Variant calling: After converting the SAM output from the alignment run to sorted BAM using sambamba [15], we then used Freebayes v1.1.0 to call variants:

```
freebayes \
    -X -u --haplotype-length 0 \
    -f ${FASTA_PATH}/hg38.fa \
    --min-mapping-quality ${MIN_MAPQ} \
    -v ${OUTPUT_VCF} \
    ${INPUT_BAM}
```

The `-X -u` arguments ensure Freebayes calls only SNVs and indels, though the indels calls were not studied here. The `--haplotype-length 0` argument ensures each SNV appears as a separate call in the VCF file, even in cases where nearby SNVs can be phased into a haplotype block. This helps make the SNVs more comparable between the Freebayes output and the Platinum calls. `-f` specifies the reference FASTA file. `--min--mapping-quality` specifies the minimum mapping quality an alignment must have to be included in the variant calling analysis. We run Freebayes repeatedly setting different values for `${MIN_MAPQ}`. We also tried running Freebayes in three other modes affecting the mapping quality threshold: (a) its default mode, (b) `--standard-filters` mode, and (c) `--use-mapping-quality`. But for none of the βs tested did the cutoffs enforced by any of those three modes yield maximal F_β. In fact, the thresholds yielding maximal F_β were always in the range 2–5.

The above command was run twice for every mapping quality threshold: once with `${INPUT_BAM}` is set to the sorted BAM derived from the `input.sam` file, and once with with `${INPUT_BAM}` set to the sorted BAM derived from `input.sam`.

Variant filtering: We applied two filters to the variant calls output by Freebayes. First, we eliminated variant calls with spuriously high coverage, as suggested in prior work [10]. Spuriously high coverage tends to indicate the presence of a copy number change that confounds variant calling. This was accomplished with a simple `awk` script that removed VCF records with depth (as indicated by the `DP` field of the `INFO` column) at

least four poisson standard deviations above the mean. Mean depth of coverage was calculated to be 52.79 using `samtools flatstat`, so the filter removed variants with depth greater than 82. This script, and all others used for these experiments, can be found at `https://github.com/BenLangmead/qtip-experiments/tree/v1.6.1`.

Next, we filtered the Freebayes output to eliminate variant calls outside genomics regions deemed "high confidence" by the Platinum Genomes [4] project. We used the `vcfintersect` tool from the `vcflib` (`https://github.com/vcflib/vcflib`) collection; the specific command was:

```
vcfintersect -b ${PLATINUM_BED} ${VCF}
```

Where `${VCF}` was the file produced by Freebayes and `${PLATINUM_BED}` was a version of the Platinum Genomes high-confidence region `bed` file obtained from the Platinum Genomes FTP site (`ftp://ussd-ftp.illumina.com/2016-1.0/hg38/small_variants/`). We first preprocessed the `${PLATINUM_BED}` file to retain only regions in chromosomes 1–22 and X.

F scores: To obtain F-scores we compiled two `roc` files, one for the variant calls using the aligner-predicted mapping qualities and one for the calls predicted by Qtip. A `roc` file summarizes the number of true positives, false positives, and false negatives at all possible `QUAL` cutoffs. The `QUAL` field of the VCF file gives Freebayes' "genotype confidence." The ROCs were obtained by running `vcfroc`, a tool in the `vcflib` (`https://github.com/vcflib/vcflib`) collection. The `vcfroc` command was:

```
vcfroc \
    --truth-vcf ${PLATINUM_VCF} \
    --reference ${FASTA_PATH}/hg38.fa \
    ${VCF_FN} > ${ROC_FN}
```

Finding the maximal F scores: All of the above experiments were carried out for all mapping-quality thresholds and for both the original and the Qtip-predicted mapping qualities. For the original mapping qualities and given a particular β, we calculated maximal F_β by evaluating F_β:

$$F_\beta = (1+\beta^2) \cdot \frac{precision \cdot recall}{(\beta^2 \cdot precision) + recall}$$

For every possible combination of mapping-quality and genotype-quality threshold. We then reported the maximal F_β.

Note 10 Setting the number of tandem reads

Qtip has parameters that control the minimum number of tandem reads or pairs of each category (*conc*, *disc*, *bad-end* and *unp*) to generate. The default number of training reads/pairs generated for each category is $45 \cdot \sqrt{x}$ where x is the number of input alignments of that category. Both the scaling factor and the function are configurable via Qtip's `--sim-function` and `--sim-factor` parameters.

We conducted a set of simulation experiments on a few selected combinations of functions and coefficients. We used Mason to simulate two sets of 50M reads, one unpaired set and one paried-end. Both sets used 100 nt reads, with paired-end reads having simulated fragments lengths mostly between 200 and 400 nt, per the strategy described in Note 4. We tried the following policies for setting a target number of tandem alignments:

1. $15 \cdot \sqrt{x}$
2. $30 \cdot \sqrt{x}$
3. $45 \cdot \sqrt{x}$
4. $0.01 \cdot x$
5. $0.03 \cdot x$
6. $0.05 \cdot x$
7. $50,000$
8. $100,000$

We say that these policies set a "target" number of alignments because the actual tandem simulation procedure could result in fewer or more alignments. This is because (a) not every tandem read will align successfully, (b) the target is inflated somewhat prior to simulation in order to account for alignment failures, and (c) the tandem simulator actually conducts a large number of binomial draws; the total across all the draws will be very close to, but not necessarily equal to, the inflated target.

We tried each of these policies for each of the following numbers of input reads:

1. 1,000,000
2. 5,000,000
3. 10,000,000
4. 50,000,000

The script used to drive these experiments is present in the `qtip-experiments` repository as experiments/simulated_reads/train_series.sh.

As seen in Figure S3, the formulae result in similar levels accuracy as the number of input reads grows to 50,000,000, with an RCE spread of less than 2 in the unpaired case and less than 1 in the paired-end case. Setting the target to $50,000$ performs worst at at the highest numbers of input reads, suggesting the constant functions tend to perform poorly as the number of input alignment grows large. The linear functions (reddish lines) and those that grow as a function of the square root of the number of input alignments (greenish lines) perform similarly, though the $0.01 \cdot x$ target performs poorly with only 1,000,000 input reads.

Ultimately, we prefer the formulae that grow with the square root of the number of input reads, as they represent a compromise between constant functions that perform poorly at higher numbers and linear functions that require large amounts of training data at larger input sizes. The square-root functions yield comparable RCE to the linear functions. Qtip's default formula is $45 \cdot \sqrt{x}$.

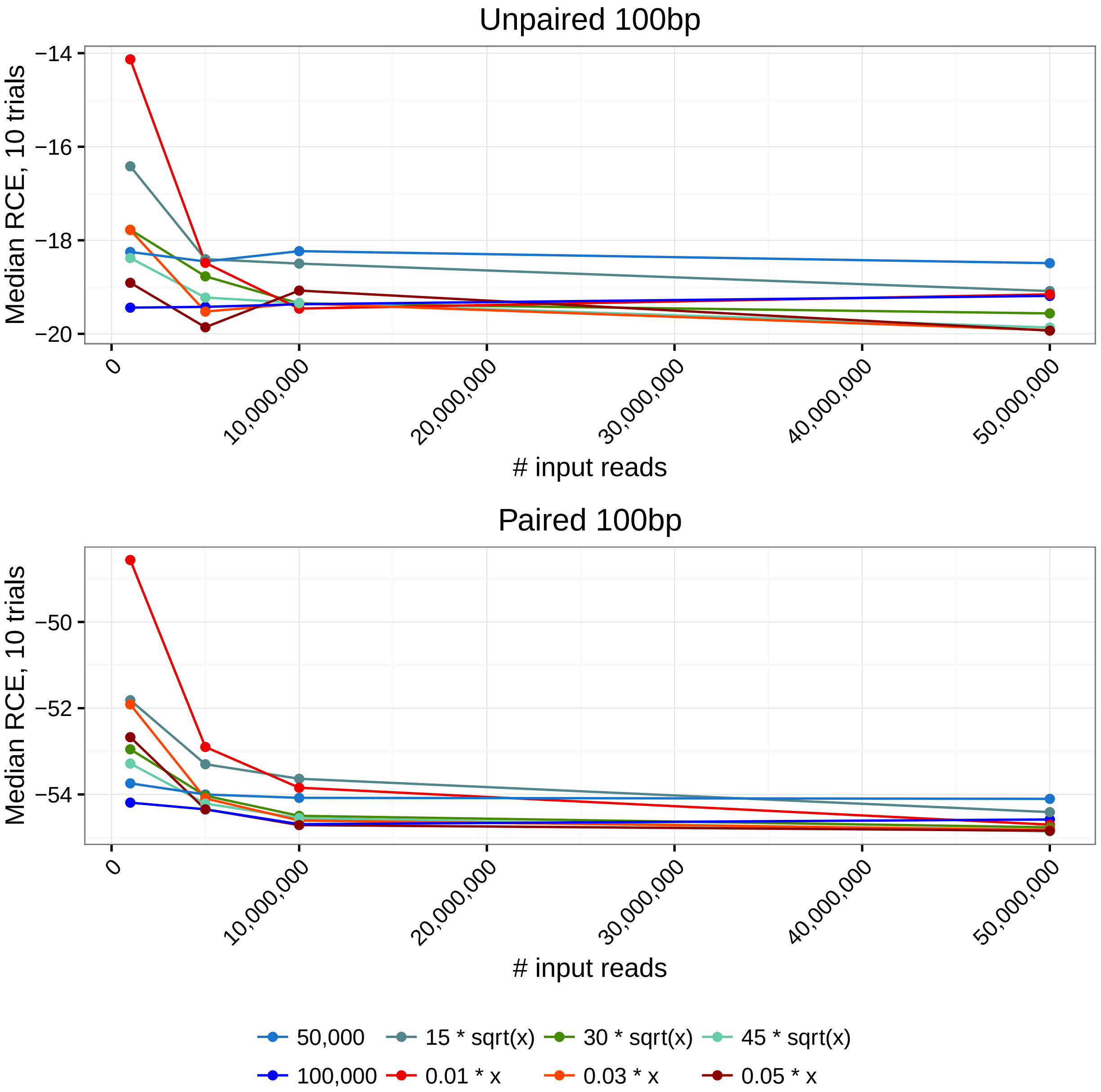

Figure S3: Series of experiments showing how formulas for setting the target number of tandem alignments affect accuracy, measured as the median relative change in squared error across 10 trials.

Note 11 Input modeling and tandem simulation

Scanning the input. The construct the input model, Qtip using reservoir sampling to obtain an appropriate-sized subset of the input alignments. It does this over the course of a single scan through the input alignments. For paired-end alignments, Qtip's scan matches up the two ends so that they can be considered together here. Reads that fail to align are ignored, as are non-primary alignments,. During the scan, Qtip distinguishes between the four different categories of alignment: *conc*, *disc*, *unp* and *bad-end*. A separate reservoir sampler is used for each category. The maximum number of alignments sampled in a category can be configured with Qtip's `--input-model-size` parameter (default: 30,000).

Templates. Every category of template contains at least the following fields:

- Score
- Read length
- Strand
- Quality string
- Edit transcript

Alignment score is copied from the `AS:i` SAM field. Read length is easily calculated from the SAM/BAM `SEQ` field. Strand is inferred from the SAM `FLAG` field. Quality string is copied from the SAM `QUAL` field.

Edit transcript is inferred from a different combination of SAM fields depending on the aligner used. For Bowtie 2 and BWA-MEM , it is inferred from the `CIGAR` and `MD:Z` fields. SNAP does not report the `MD:Z` field, and the CIGAR field alone is not always sufficient to determine where mismatches are located. However, SNAP can optionally print a CIGAR string that does contain sufficient information to determine where mismatches are located (the `-=` parameter), so when running SNAP from Qtip, that parameter is always specified.

When that parameter is specified, the edit transcript can be inferred from the CIGAR field alone.

The 5 fields listed in the previous subsection are sufficient for representing an unpaired read template. A bad-end template includes two additional fields:

- Whether aligned end is end 1 or 2
- Length of the (unaligned) opposite end

These are inferred from the SAM `FLAG` and `SEQ` fields for the opposite end.

A *conc* template contains all of the fields listed above plus these additional fields:

- Score of the opposite end
- Sum of scores of the two ends
- Strand of opposite end
- Quality string of opposite end
- Edit transcript of opposite end
- Whether end 1 aligns to the left of end 2 with respect to the reference
- Fragment length

Most of these additional fields are inferred straightforwardly from the SAM records for the two ends as described previously. Which end aligns upstream is determined by examining the SAM `POS` field for the two records. Fragment length is inferred from the SAM `TLEN` field.

A *disc* template is identical to a *conc* template. Note, though, that the fragment length field may not be interpretable for a discordantly aligned pair, since the two discordantly-aligned ends can align very far apart or even to different chromosomes.

Handling soft clipping. Input alignments with soft-clipped bases (`S` CIGAR operation) pose a problem for our templates: because the soft-clipped bases failed to align, we do not know how to represent them in the edit transcript. Simply excluding those bases from the

edit transcript – effectively trimming them from the tandem read – is not desirable. We would like the tandem reads to mimic the input reads in all important ways, including in the prevalence of soft-clipping.

One solution is to, for each instance of soft clipping, extract the corresponding stretch of reference bases and perform post-facto alignment of the soft-clipped bases. The alignment would likely be poor (hence the soft clipping), but would yield informative values for the edit transcript. This is slow in practice, however, since it requires that we extract a arbitrary stretch of the reference genome – requiring disk head movement in many cases – for each instance of soft clipping.

Instead, Qtip represents soft-clipped bases with special `S` characters in the edit transcript. When simulating the read, Qtip simply inserts a random base into each soft-clipped position. Advantages of this approach are: (a) it is fast and simple, requiring no additional alignment, and (b) random bases are a reasonable proxy for soft-clipped bases since' they are unlikely to align well to the reference, and so will likely be clipped. The disadvantage is that there are no guarantees about how successfully the random bases will act as a proxy for the soft-clipped bases. For example, the random bases could, by coincidence, match the reference genome well enough that they align without any soft clipping, or with less soft clipping than was needed for the corresponding input read. This causes the tandem reads to depart somewhat from the character of the input reads, though Qtip's good performance on local read aligners suggests that the effect is likely minor.

Simulating from templates. Qtip uses the input models to create the tandem read set in the following way. The user specifies the location of the reference genome in FASTA format to Qtip. Qtip iterates through overlapping windows (*chunks*) of the reference genome. Each chunk consists of $B+O$ contiguous bases, where the base length B is 100,000 by default. We discuss later how the overlap length O is determined. The O bases at the end of

the $B - O$ sized chunk will appear again at the beginning of the following chunk, except in cases where, for example, we reach the end of a chromosome.

Overlaps between chunks are to ensure all possible read starting positions are equally probable. When a read is simulated, the read's leftmost base must fall within the first B bases of the current chunk. The read's leftmost base may not fall within the final O bases (though those bases will appear again at the beginning of the following chunk).

For each chunk and each alignment category, Qtip calculates the number of reads to simulate according to a binomial draw with $n = B$, the number of possible starting positions for the fragment, and $p = \frac{G}{n}$ where G is the genome length and n is the number of reads of that category we aim to simulate in total. G is estimated from the FASTA file sizes, and n is set according to the `--sim-*` arguments passed to Qtip.

Each unpaired read is then simulated by (1) picking a template of the appropriate type uniformly at random from among those sampled, (2) picking a leftmost read position from among the first B positions of the chunk uniformly at random, (3) extracting an appropriate-length substring of the reference genome starting at the chosen position, (4) mutating the extracted reference substring according to the strand and edit transcript information in the template, (5) setting the read name equal to a special string that encodes the read's true point of origin, (6) setting the read quality string equal to the quality string in the template, and finally (7) printing the read to a FASTQ file in preparation for the tandem alignment step.

A bad-end read is simulated similarly except that a dummy opposite end is synthesized by generating a random nucleotide sequence. The dummy opposite end is virtually guaranteed not to align, mimicking the situation for the input pair.

Paired-end reads are simulated similarly except that when extracting the substring from the reference genome, a substring corresponding to the entire fragment is extracted. In the case of a *conc* pair, fragment length is dictated by the length given in the template. In the case of a *disc* pair, fragment length is set to a pre-determined value larger

than the largest concordant fragment length simulated. This is configurable via Qtip's `--max-allowed-fraglen` parameter. Once a fragment substring is extracted, read sequences are extracted from either end of the fragment, choosing the upstream end and the orientations according to the template.

In order to simulate a long fragment, the entire fragment must be present in some chunk returned by the FASTA parser. To guarantee this will be the case, Qtip sets the overlap length O equal to the maximum fragment or read length in any of the input models.

Aligning tandem reads. After tandem reads are simulated, they are aligned (step 4) using the same read aligner and the same alignment parameters as were used to align the input reads in step 1. However, circumstances can cause the tandem alignments to depart from the template.

First, a tandem read may simply fail to align, decreasing the number of Qtip's training examples by one. This is relatively rare, since the templates themselves correspond to reads that aligned successfully, and are therefore unlikely to run seriously afoul of the aligner heuristics. However, the tandem read might be extracted from a qualitatively different genomic region than the original read. For example, the tandem read might be drawn from a repeat, whereas the original read was not. This increases the chance that, e.g., the aligner might stop early before finding a valid alignment for the tandem read.

Second, the tandem read might align successfully, but with a different alignment score and/or edit transcript from those in the template. E.g., if the tandem read is drawn from a repetitive portion of the genome, the edits induced by the transcript might cause the read to align with higher alignment score elsewhere. Alternately, the aligner might fail to align the read to its true point of origin and instead align it such that its score is lower than the template score. We so no strong evidence that Qtip's predictions are systematically biased in either direction, either producing tandem alignments with overall higher or

overall lower scores than the input alignments.

Discussion. We described a method for deriving input models from aligned reads. We also described a method for using the input models, together with the reference genome, to simulate a collection of tandem reads that mimic the input reads in key ways. The method has several advantages: (a) it is simple, not requiring an external read simulator, (b) input models are built during a single pass over the input alignments, (c) reads are simulated during a single pass over the reference genome, (d) since the input alignments are scanned one- or two-at-a-time, and since only a single chunk of the reference genome considered at a time during simulation, the added memory footprint is low.

It should also be noted that, because read sequences are drawn randomly from across the genome and are matched with templates randomly, the tandem reads taken as a whole are not consistent with any particular genome sequence. That is, the reads will not generally agree with each on, e.g., where any SNPs or other variants are located with respect to the reference genome. But since the read aligners themselves consider each read independently and one at a time, this should not affect the performance of the mapping quality model trained in step 6.

Note 12 Feature importances

We studied the feature importances reported by the `RandomForestRegressor` model for the simulation experiments described in the main text in Figure 2 and Table 2. Feature importances are calculated by `scikit-learn` and included in the object representing the fit model. Importances range from 0 to 1, with higher importance indicating the feature tends to appear higher in the decision trees.

Recall that a separate model is trained for each of the four categories of alignment: `conc`, `disc`, `bad-end`, and `unp`. The first three are relevant for the paired-end simulations and the last for unpaired simulations. We plot importances for the 6 features with the highest average importance across all trials and reference genomes. The boxplots show the importances across the 10 trials.

Recall that the random forest model is indifferent to scale. Some of the features described below should intuitively have a positive correlation with mapping quality (e.g. Score diff) while others should have a negative correlation (e.g. % repetitive). The random forest is not predisposed to one or the other.

Bowtie 2 Figure S4 shows results for the Bowtie 2 experiments for 100 nt reads for all reference genomes and models. Figure S5 shows the same for 250 nt reads. The features mentioned in those plots are:

- Best score: Alignment score of the reported (best) alignment.
- Score diff: Score of best alignment minus score of second-best alignment, using the configured scoring scheme. If no second-best alignment is found, this is set to a value larger than the largest observed score difference.
- Score diff other: Like Score diff but for the opposite rather than the current end of the paired-end alignment.

- Conc score diff: Score of best concordant paired-end alignment minus score of second-best concordant paired-end alignment, using the configured scoring scheme. The score of a concordant alignment is calculated as the sum of the alignment scores of both ends. If no second-best concordant paired-end alignment is found, this is set to a value larger than the largest observed difference.
- Score diff other: Same as Score diff but for the opposite rather than the current end of the paired-end alignment.
- Edit diff: Edit distance of best alignment minus edit distance of second-best alignment, ignoring the configured scoring scheme. If no second-best alignment is found, this is set to a value larger than the largest observed edit distance difference.
- Edit diff other: Same as Edit diff but for the opposite rather than the current end of the paired-end alignment.
- Conc edit diff: Edit distance of best concordant paired-end alignment minus edit distance of second-best concordant paired-end alignment, ignoring the configured scoring scheme. The edit distance of a concordant alignment is calculated as the sum of the edit distances of both ends. If no second-best concordant paired-end alignment is found, this is set to a value larger than the largest observed difference.
- % unique stranded: Fraction of seeds from the same strand as the reported alignment that matched the reference in exactly one location.
- % unique stranded other: Same as % unique stranded but for the opposite rather than the current end of the paired-end alignment.
- % unique: Fraction of seeds that matched the reference in exactly one location, regardless of seed's strand.
- % repetitive: Fraction of seeds that matched the reference in more than one location, regardless of seed's strand.
- Avg seed hits: Average number of times a seed matched the reference.
- Avg seed hits stranded: The average number of times a seed from the same strand

as the reported alignment matched the reference.

- Frag length: Inferred fragment length as reported in the SAM `TLEN` field.

BWA-MEM Figure S6 shows results for the BWA-MEM experiments for 100 nt reads for all reference genomes and models. Figure S7 shows the same for 250 nt reads. The features mentioned in those plots are:

- Best score: Alignment score of the reported (best) alignment.
- Score diff: Score of best alignment minus score of second-best alignment, using the configured scoring scheme.
- Score diff o: Like Score diff but for allows the second-best alignment to be redundant with the first.
- Score diff other: Like Score diff but for the opposite rather than the current end of the paired-end alignment.
- Score diff o other: Like Score diff o but for the opposite rather than the current end of the paired-end alignment.
- Conc score diff: Score of best concordant paired-end alignment minus score of second-best alignment, using the configured scoring scheme. The score of a concordant alignment is calculated as the sum of the alignment scores of both ends.
- # tied for 2nd: Number of alignments known to be tied at the second-best alignment score.
- # tied for 2nd o: Like # tied for second but for allows the second-best alignment to be redundant with the first.
- # tied for 2nd o other: Like # tied for second o but for the opposite rather than the current end of the paired-end alignment.
- # conc tied for second: Number of concordant alignments known to be tied at the

second-best alignment score. The score of a concordant alignment is calculated as the sum of the alignment scores of both ends.

- Seed coverage: Fraction of read length covered by aligning seeds.
- % repetitive: Fraction of seeds that matched the reference in more than one location, regardless of seed's strand.
- Frag length: Inferred fragment length as reported in the SAM `TLEN` field.

SNAP Figure S8 shows results for the SNAP experiments for 100 nt reads for all reference genomes and models. Figure S9 shows the same for 250 nt reads. The features mentioned in those plots are:

- Prob best: SNAP's estimate of the probability the best alignment is correct.
- Prob best other: Like Prob best but for the opposite rather than the current end of the paired-end
- Score diff: Edit distance of best alignment minus edit distance of second-best concordant paired-end alignment, ignoring the configured scoring scheme. (Note: SNAP's scoring scheme is edit distance, so "edit distance" and "score" are used interchangeably here.) If no second-best alignment was found, this is set to a value just smaller than the most negative observed difference.
- Score diff other: Like Score diff but for the opposite rather than the current end of the paired-end
- Min hits: The minimum number of matches for any seed, regardless of strand.
- Min hits other: Like Min hits other but for the opposite rather than the current end of the paired-end
- Min hits strand: The minimum number of matches for any seed taken from the same strand as the reported alignment.

- Min hits strand other: Like Min hits strand but for the opposite rather than the current end of the paired-end alignment.
- Pop seeds skip: Number of seeds ignored because they matched the reference too many times.
- Hits per lookup: Average number of seed hits per index query.
- Hash misses: Number of index queries that returned 0 seed hits.
- Frag length: Inferred fragment length as reported in the SAM `TLEN` field.

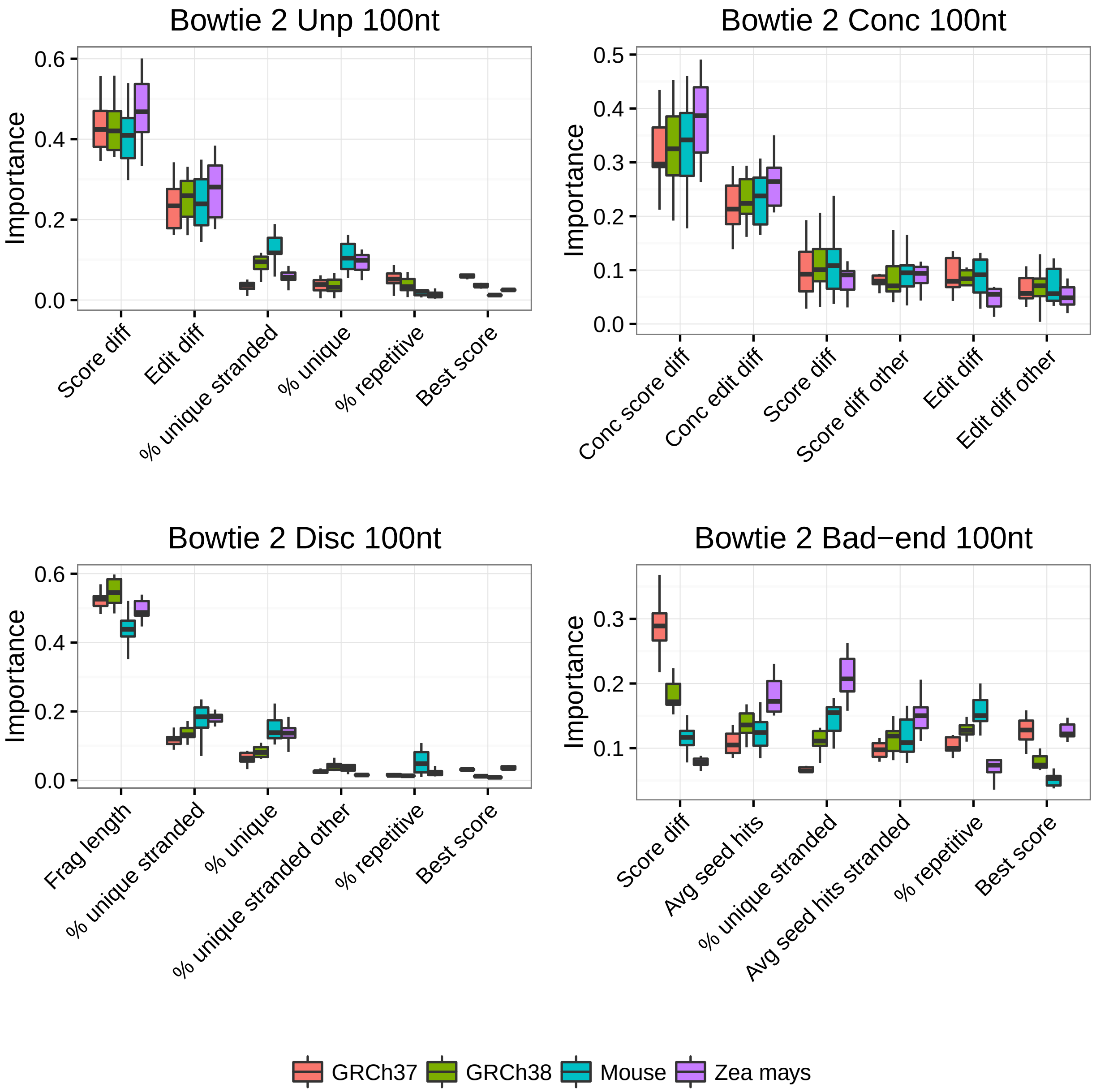

Figure S4: Feature importances for Bowtie 2 experiments on 100 nt reads for all reference genomes and models. Plotted are the 6 features with greatest average importance across all trials and reference genomes. Boxplots show importance measurements across 10 trials. The model for the unpaired simulation is labeled "Unp" and models for the paired-end simulations are labeled "Conc," "Disc" and "Bad-end." A more detailed description of the features is given in Note 12.

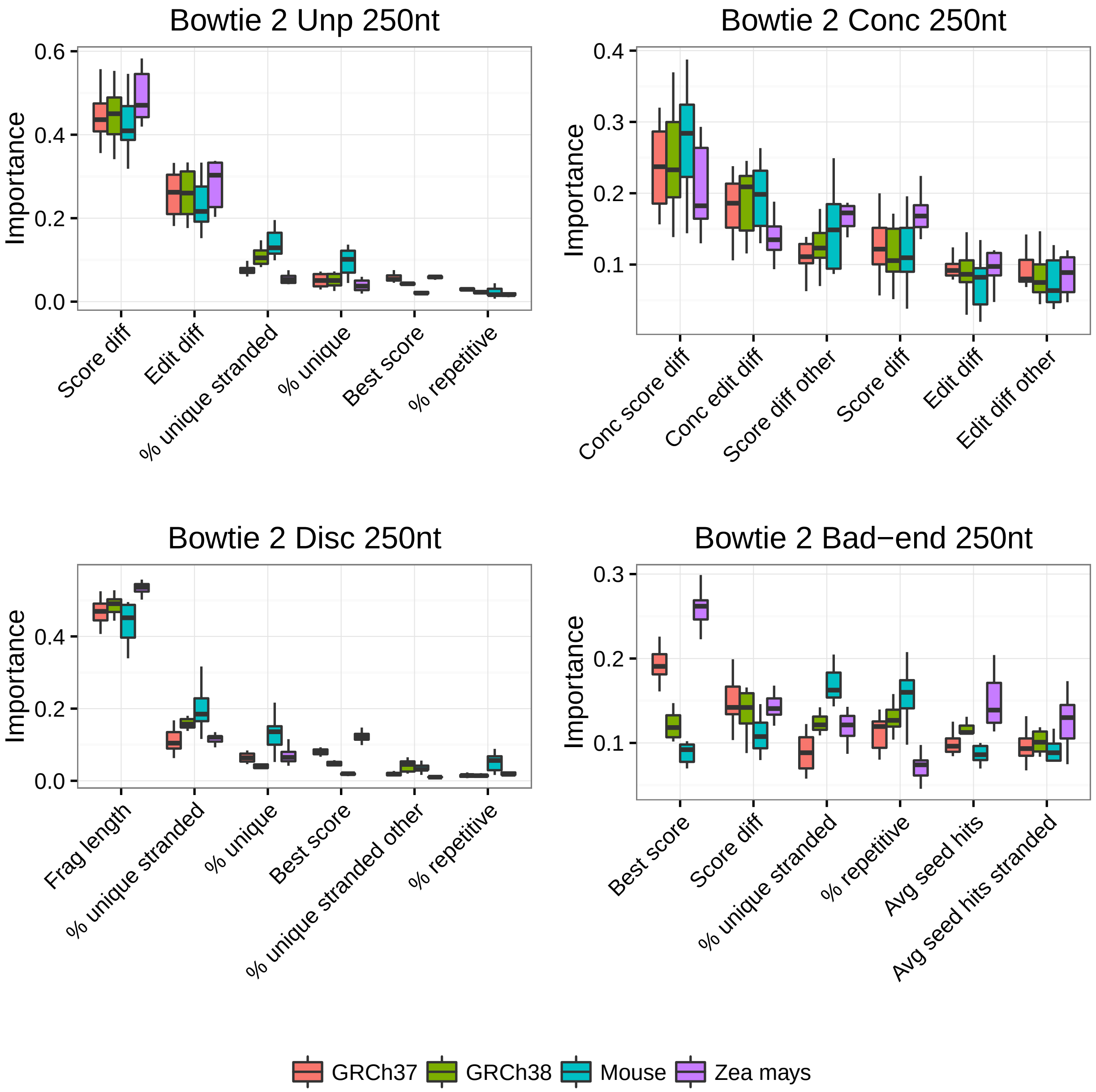

Figure S5: Feature importances for Bowtie 2 experiments on 250 nt reads for all reference genomes and models. Plotted are the 6 features with greatest average importance across all trials and reference genomes. Boxplots show importance measurements across 10 trials. The model for the unpaired simulation is labeled "Unp" and models for the paired-end simulations are labeled "Conc," "Disc" and "Bad-end." A more detailed description of the features is given in Note 12.

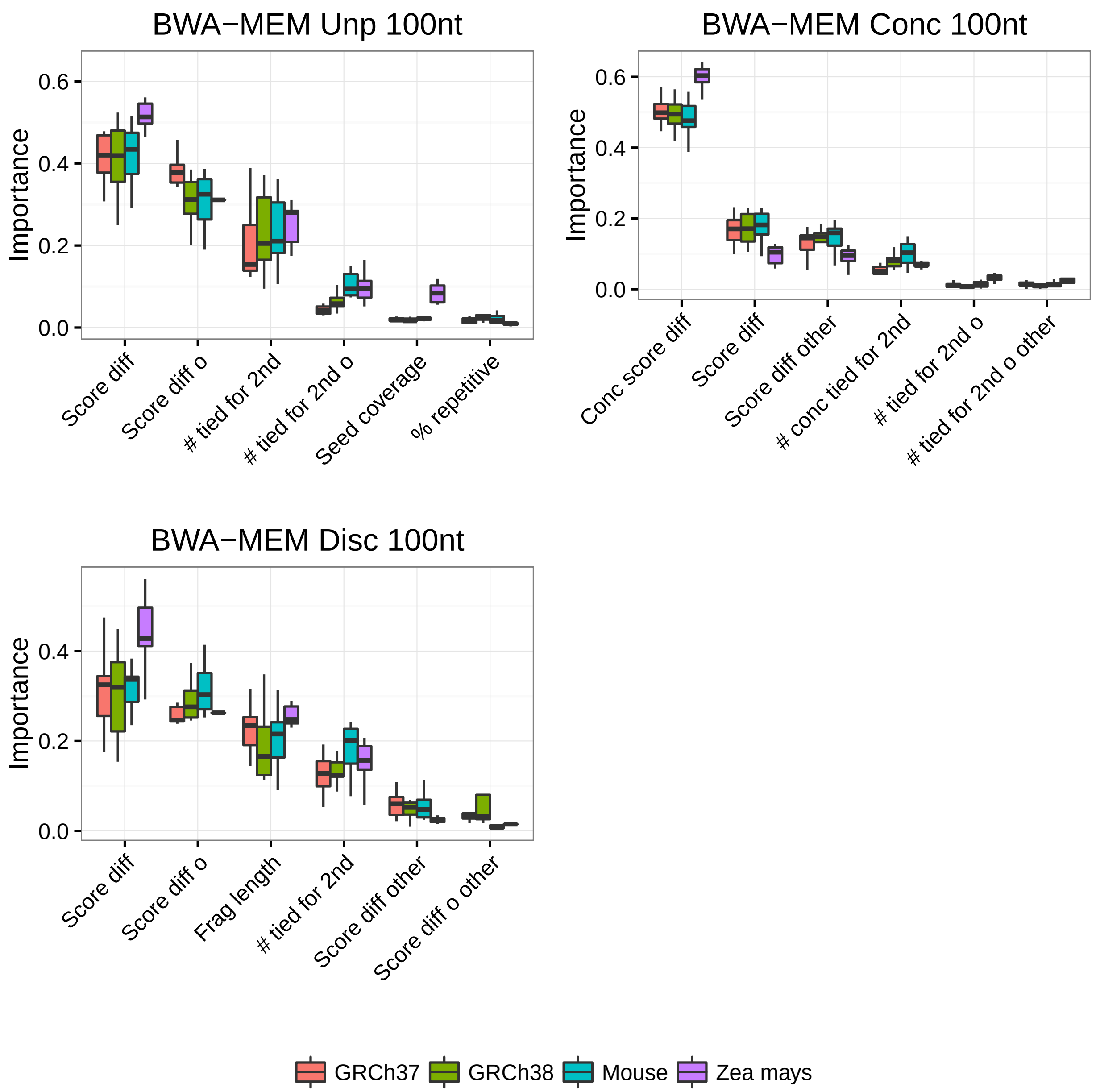

Figure S6: Feature importances for BWA-MEM experiments on 100 nt reads for all reference genomes and models. Plotted are the 6 features with greatest average importance across all trials and reference genomes. Boxplots show importance measurements across 10 trials. The model for the unpaired simulation is labeled "Unp" and models for the paired-end simulations are labeled "Conc" and "Disc." "Bad-end" is absent because BWA-MEM does not report any instances where only one end of a pair aligns. A more detailed description of the features is given in Note 12.

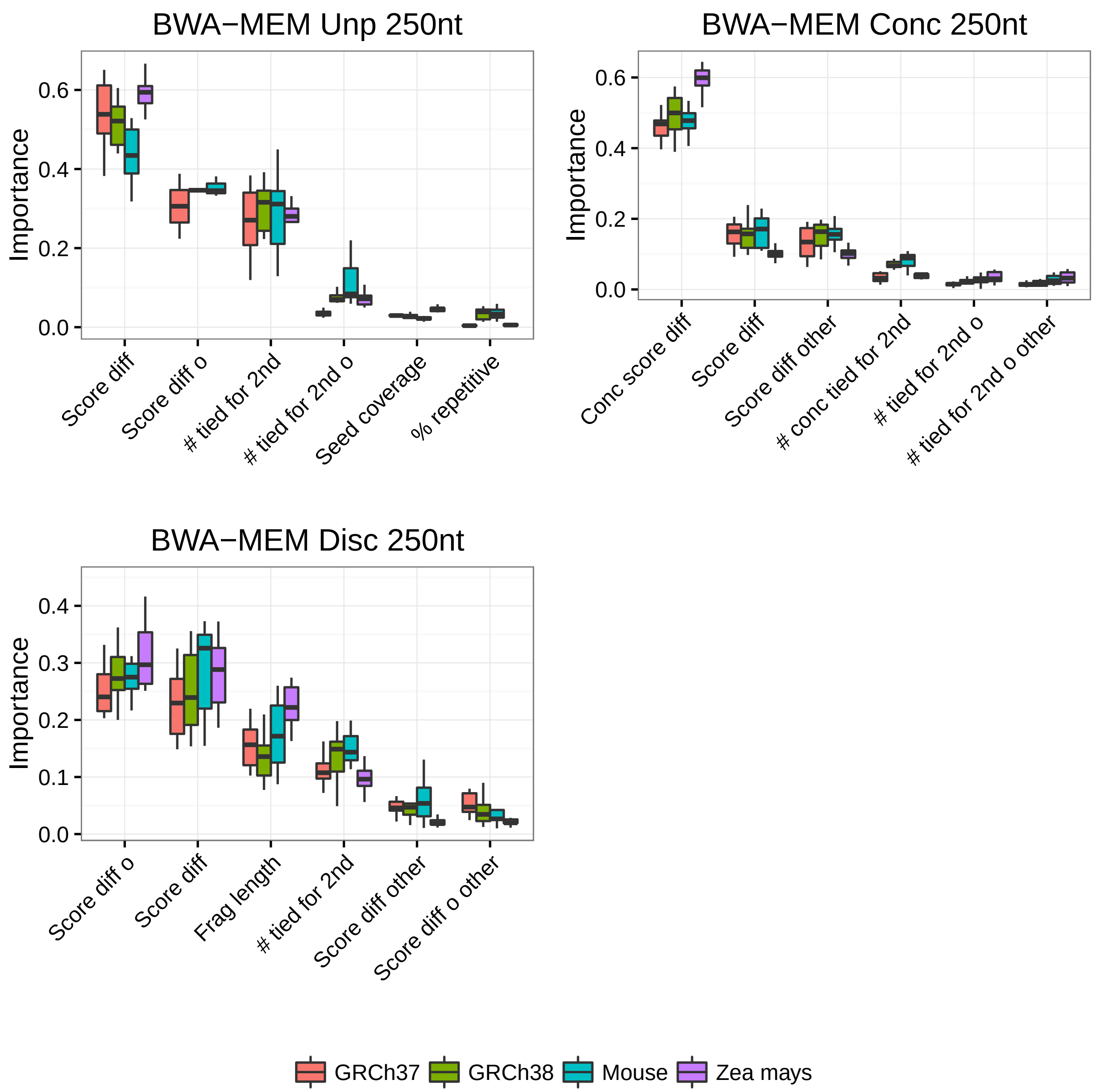

Figure S7: Feature importances for BWA-MEM experiments on 250 nt reads for all reference genomes and models. Plotted are the 6 features with greatest average importance across all trials and reference genomes. Boxplots show importance measurements across 10 trials. The model for the unpaired simulation is labeled “Unp” and models for the paired-end simulations are labeled “Conc” and “Disc.” “Bad-end” is absent because BWA-MEM does not report any instances where only one end of a pair aligns. A more detailed description of the features is given in Note 12.

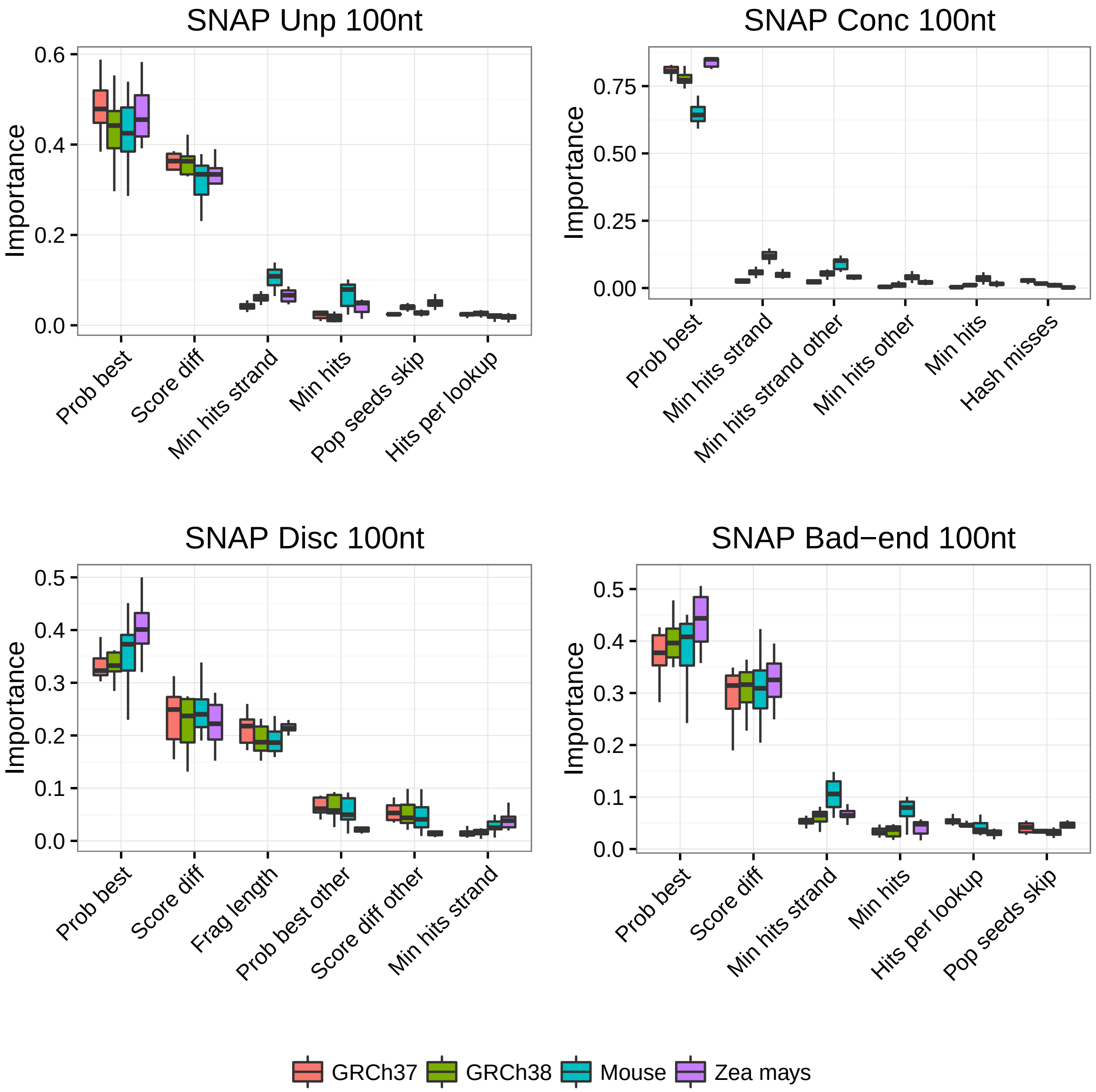

Figure S8: Feature importances for SNAP experiments on 100 nt reads for all reference genomes and models. Plotted are the 6 features with greatest average importance across all trials and reference genomes. Boxplots show importance measurements across 10 trials. The model for the unpaired simulation is labeled "Unp" and models for the paired-end simulations are labeled "Conc," "Disc" and "Bad-end." A more detailed description of the features is given in Note 12.

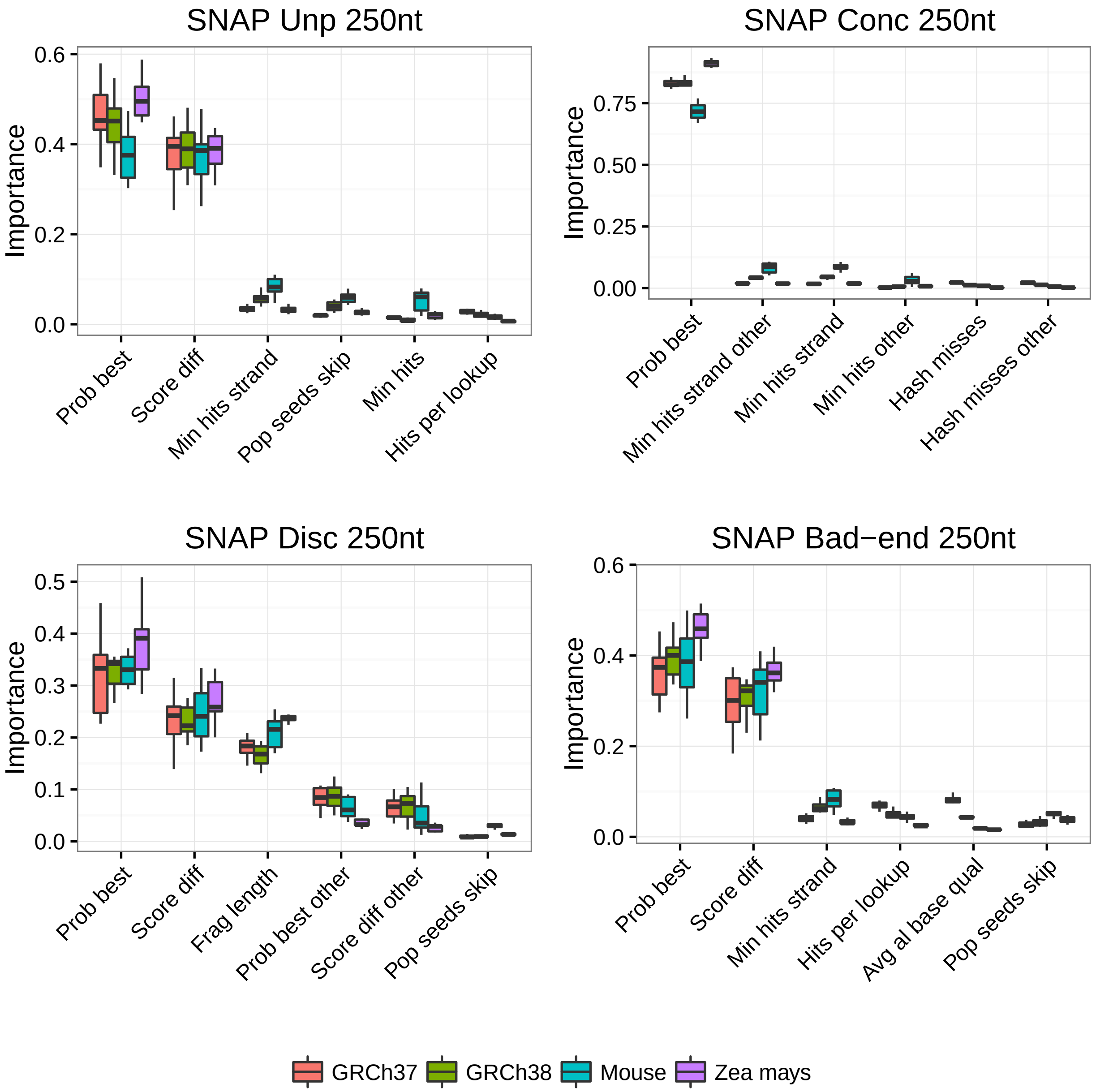

Figure S9: Feature importances for SNAP experiments on 250 nt reads for all reference genomes and models. Plotted are the 6 features with greatest average importance across all trials and reference genomes. Boxplots show importance measurements across 10 trials. The model for the unpaired simulation is labeled "Unp" and models for the paired-end simulations are labeled "Conc," "Disc" and "Bad-end." A more detailed description of the features is given in Note 12.

Note 13 Mapping-quality prediction model

Random forest model Qtip uses the scikit-learn Python module [14] to build its mapping quality prediction model. Specifically Qtip uses scikit-learn's `RandomForestRegressor`, a model consisting of an ensemble of decision trees. Each tree is trained on a bootstrap sample of the training data, and each tree contributes a "vote" as to the probability the given alignment is correct. The final prediction is an average of all trees' votes.

Random forests have many advantages in this setting. First, decision tree training is not sensitive to how features are scaled. When adding a new feature, an aligner author can simply report the data as-is, without first having to consider whether log scaling, or any other monotone scaling, is needed to bring the feature into agreement with other features. This is a major advantage over scale-sensitive models like support vector machines or neural networks.

Second, random forests are efficient. They have few hyperparameters, making the training process simpler and faster. E.g., the SNAP aligner takes over 2 hours to align the paired-end ERR050083 dataset, whereas the resulting random forests take about 2 minutes to train. This is a key advantage over, e.g., neural networks. Having said that, the prediction step – step 7 – is the single biggest contributor to Qtip's computational overhead. We plan to explore whether using a different random forest implementation could increase prediction throughput.

Finally, after a `RandomForestRegressor` model is fit, it optionally reports feature importances. A feature's importance is essentially the degree to which splitting on the feature helps to separate positive from negative training examples.

Hyperparameters Parameters governing the size, shape and splitting behavior of the random forest, often called hyperparameters, have to be chosen at model fitting time. For this model, the relevant parameters are:

- Number of decision trees in the forest. Set to 30 by default in Qtip, but adjustable using the `--num-trees` parameter.
- Maximum number of leaf nodes per decision tree. Set to 35 by default in Qtip, but adjustable using the `--max-leaf-nodes` parameter.
- Maximum number of features to split on at each internal decision tree node. This is expressed as the fraction of the total number of features, and it's allowed to vary from 0.1 to 0.45 by default. It is adjustable using the `--max-features` parameter.

As mentioned, the `--max-features` hyperparameter is not fixed. In fact, all three hyperparameters can be configured to take values in a range; e.g., specifying the parameter `--num-trees 30,35,40,45,50` will allow `--num-trees` to take on any of the specified values. For each hyperparameter allowed to vary in this way, a particular value is chosen at model-training time via a hyperparameter fitting procedure that performs hill-climbing with a configurable numeric tolerance (`--optimization-tolerance`). The hyperparameters selected are those that yield the best out-of-bag score after training. The out-of-bag score is calculated and by the `RandomForestRegressor` model. As an alternative to the out-of-bag score, cross validation can be used instead (via Qtip's `--no-oob` parameter).

Alternate models Qtip can optionally be configured to use the `ExtraTreesRegressor` class, which implements a variant on random forests called extremely randomized trees [6]. The hyperparameters are defined, configured, and fit in exactly the same manner as for the `RandomForestRegressor`.

Forming the training and test matrices The test matrix is a matrix where each row is an input alignment and each column is a feature. Elements contain feature data. In the case of a paired-end alignment, the two ends appear as separate rows in the matrix. The training matrix is similar, except that the rows are tandem alignments rather than input

alignments. For each alignment category, Qtip builds a training matrix and uses it to train the model (step 6). Qtip uses the model to predict mapping quality for the input alignments (step 7).

As mentioned, the columns of the matrices are features and the rows are alignments. Qtip might simplify the matrix in certain ways. For example, if all elements in a column are identical in the training matrix, that column is removed from both the training and test matrices. Also, if two columns of the training matrix are identical, the rightmost of the two columns is removed from both the training and test matrices.

To maintain a low memory footprint, matrices are handled such that only a single fixed-sized block of consecutive rows needs to be present in memory at a time. For example, when Qtip parses test records derived from the input SAM file, the records are read in blocks, with only one block residing in memory at a time. By default, a single block contains at most 250K records. This is configurable via Qtip's `--max-rows` option. Qtip then uses the model to predict mapping qualities for the block and writes the corresponding mapping qualities to disk before deallocating the block and moving on.

Missing values As discussed in Note 6, some feature data can be "missing." For example, an important feature used in Qtip is the difference between the score of the best and second-best alignments found by the aligner. But if the aligner fails to find a second-best alignment, this feature will take the value `NA`, meaning missing or "not available." Qtip deals with `NA` values by automatically setting all of the `NA` values in the column equal to $x+1$ where x is the maximum non-`NA` value in the column. The fact that `NA`s are set to be slightly larger than the largest non-`NA` value often suggests particular way of ordering the data values for a feature. For example, in the case of the difference between the best and second-best score, it makes sense for larger differences to be represented by larger numbers, so that when an `NA` is replaced by $x+1$, this is interpreted by the model, sensibly, as a large difference.

References

[1] D. A. Benson, M. Cavanaugh, K. Clark, I. Karsch-Mizrachi, D. J. Lipman, J. Ostell, and E. W. Sayers. GenBank. *Nucleic Acids Res.*, 45(D1):D37–D42, Jan 2017.

[2] K. Berlin, S. Koren, C. S. Chin, J. P. Drake, J. M. Landolin, and A. M. Phillippy. Assembling large genomes with single-molecule sequencing and locality-sensitive hashing. *Nat. Biotechnol.*, 33(6):623–630, Jun 2015.

[3] D. M. Church, V. A. Schneider, K. M. Steinberg, M. C. Schatz, A. R. Quinlan, C. S. Chin, P. A. Kitts, B. Aken, G. T. Marth, M. M. Hoffman, J. Herrero, M. L. Mendoza, R. Durbin, and P. Flicek. Extending reference assembly models. *Genome Biol.*, 16:13, Jan 2015.

[4] Michael A Eberle, Epameinondas Fritzilas, Peter Krusche, Morten Källberg, Benjamin L Moore, Mitchell A Bekritsky, Zamin Iqbal, Han-Yu Chuang, Sean J Humphray, Aaron L Halpern, et al. A reference data set of 5.4 million phased human variants validated by genetic inheritance from sequencing a three-generation 17-member pedigree. *Genome Research*, 27(1):157–164, 2017.

[5] Otto Erlwein, Mark J Robinson, Simon Dustan, Jonathan Weber, Steve Kaye, and Myra O McClure. Dna extraction columns contaminated with murine sequences. *PLoS One*, 6(8):e23484, 2011.

[6] Pierre Geurts, Damien Ernst, and Louis Wehenkel. Extremely randomized trees. *Machine learning*, 63(1):3–42, 2006.

[7] Manuel Holtgrewe. Mason–a read simulator for second generation sequencing data. *Technical Report FU Berlin*, 2010.

[8] Weichun Huang, Leping Li, Jason R Myers, and Gabor T Marth. Art: a next-generation sequencing read simulator. *Bioinformatics*, 28(4):593–594, 2012.

[9] S. Kurtz, A. Phillippy, A. L. Delcher, M. Smoot, M. Shumway, C. Antonescu, and S. L. Salzberg. Versatile and open software for comparing large genomes. *Genome Biol.*, 5(2):R12, 2004.

[10] Heng Li. Toward better understanding of artifacts in variant calling from high-coverage samples. *Bioinformatics*, 30(20):2843–2851, Oct 2014.

[11] Richard W Lusk. Diverse and widespread contamination evident in the unmapped depths of high throughput sequencing data. *PloS one*, 9(10):e110808, 2014.

[12] M. Nattestad and M. C. Schatz. Assemblytics: a web analytics tool for the detection of variants from an assembly. *Bioinformatics*, 32(19):3021–3023, Oct 2016.

[13] Anthony O Olarerin-George and John B Hogenesch. Assessing the prevalence of mycoplasma contamination in cell culture via a survey of ncbi's rna-seq archive. *Nucleic acids research*, 43(5):2535–2542, 2015.

[14] F. Pedregosa, G. Varoquaux, A. Gramfort, V. Michel, B. Thirion, O. Grisel, M. Blondel, P. Prettenhofer, R. Weiss, V. Dubourg, J. Vanderplas, A. Passos, D. Cournapeau, M. Brucher, M. Perrot, and E. Duchesnay. Scikit-learn: Machine learning in Python. *Journal of Machine Learning Research*, 12:2825–2830, 2011.

[15] Artem Tarasov, Albert J Vilella, Edwin Cuppen, Isaac J Nijman, and Pjotr Prins. Sambamba: fast processing of ngs alignment formats. *Bioinformatics*, 31(12):2032–2034, 2015.
